# Causal relationship between gut microbiota and insulin-like growth factor 1: a bidirectional two-sample Mendelian randomization study

**DOI:** 10.3389/fcimb.2024.1406132

**Published:** 2024-09-24

**Authors:** Xuejie Zheng, Yuping Qian, Lili Wang

**Affiliations:** ^1^ Department of Pediatrics, First Affiliated Hospital of Anhui Medical University, Hefei, Anhui, China; ^2^ Department of Neonatology, Anhui Provincial Children’s Hospital, Hefei, Anhui, China

**Keywords:** gut microbiota, IGF-1, SNPs, genome-wide association studies, Mendelian randomization

## Abstract

**Background:**

The causal relationship between gut microbiota and insulin-like growth factor 1 (IGF-1) remains unclear. The purpose of this study was to explore the causal relationship between gut microbiota and IGF-1 in men and women.

**Methods:**

Single-nucleotide polymorphisms (SNPs) related to gut microbiota were derived from pooled statistics from large genome-wide association studies (GWASs) published by the MiBioGen consortium. Pooled data for IGF-1 were obtained from a large published GWAS. We conducted Mendelian randomization (MR) analysis, primarily using the inverse variance weighted (IVW) method. Additionally, we performed sensitivity analyses to enhance the robustness of our results, focusing on assessing heterogeneity and pleiotropy.

**Results:**

In forward MR analysis, 11 bacterial taxa were found to have a causal effect on IGF-1 in men; 14 bacterial taxa were found to have a causal effect on IGF-1 in women (IVW, all *P* < 0.05). After false discovery rate (FDR) correction, all bacterial traits failed to pass the FDR correction. In reverse MR analysis, IGF-1 had a causal effect on nine bacterial taxa in men and two bacterial taxa in women respectively (IVW, all *P* < 0.05). After FDR correction, the causal effect of IGF-1 on order Actinomycetales (*P_FDR_* = 0.049) remains in men. The robustness of the IVW results was further confirmed after heterogeneity and pleiotropy analysis.

**Conclusion:**

Our study demonstrates a bidirectional causal link between the gut microbiota and IGF-1, in both men and women.

## Introduction

The gut microbial system consists of at least 100 trillion bacteria and archaea in the human gastrointestinal tract and is the largest ecosystem in the human body. In addition to the large number of microorganisms, the gut microbiota has 100 times more genes than the human genome. It includes more than 1,000 bacterial species in the human colon alone and consists of at least 160 bacterial species in each individual (Qin et al., 2010; Sommer and Bäckhed, 2013; Fukuda and Ohno, 2014). An increasing number of studies have concluded that either the type or the amount of gut microbiota may influence the development of disease (Tang et al., 2017; Busnelli et al., 2019) and have even considered the gut microbiota as a separate endocrine organ (Possemiers et al., 2011). It is now recognized that the gut microbiota is closely related to host health and that the two are interdependent, affecting the host’s digestive function, intestinal permeability, endocrine system, resistance to foreign pathogens, and immune stimulation (Blumberg and Powrie, 2012). Studies have reported that the gut microbiome has a clear role in the development of type 2 diabetes and in the treatment of obesity (Ley et al., 2005; Larsen et al., 2010; Karlsson et al., 2013; Zhang et al., 2013; Kreznar et al., 2017; Kootte et al., 2017). In addition, extensive associations between the gut microbiome and other complex traits have been revealed by related studies (Kurilshikov et al., 2021); however, the causal relationship, defined as a direct cause-and-effect relationship where one variable directly influences another, between these associations is currently unknown.

Insulin-like growth factor 1 (IGF-1), as a key growth factor as bone growth, may also be closely related to the gut microbiota. Two complementary studies in the invertebrate *Drosophila melanogaster* provide preliminary evidence that the microbiota can influence host IGF-1 production (Shin et al., 2011; Storelli et al., 2011). Subsequent studies from multiple experiments have demonstrated that the microbiota also affects levels of IGF-1 and its orthologs in chicken, zebrafish, and mice (Avella et al., 2012; Kareem et al., 2016; Schwarzer et al., 2016; Yan et al., 2016). It should be emphasized that, in the mouse study, mice with intact gut microbiota (conventionally raised) had significantly higher IGF-1 levels than germ-free mice (Schwarzer et al., 2016). In addition, serum IGF-1 levels in adult germ-free mice reconstituted with conventional microbiota were significantly higher than those in littermates that continued to remain germ-free (Yan et al., 2016). Additionally, recent studies have indicated a connection between the GH–IGF-1 axis and the gut microbiome (Jensen et al., 2020a, 2020b). However, most of the current studies are observational and cannot provide further evidence of a causal relationship between the gut microbiota and IGF-1.

A causal relationship implies a direct influence of one variable on another, which is critical for understanding the underlying mechanisms. Mendelian randomization (MR) is a robust and effective method (Emdin et al., 2017) that uses genetic variants [single-nucleotide polymorphisms (SNPs)] as instrumental variables (IVs) to explore the causal effects of the gut microbiome on IGF-1. MR leverages the random assortment of genes at conception, which mimics the randomization process in controlled trials, thus helping to infer causality rather than mere association. Many previous studies have described the principles of MR and its reliability (Smith and Ebrahim, 2003; Ghavami et al., 2021). MR has been widely used to explore the causal relationship between exposure and disease and has been used several times in studies of the relationship between gut microbiota and disease (Yang et al., 2022; He et al., 2023; Xue et al., 2023).

The goals of this study were to explore the possible causal relationship between gut microbiota and IGF-1 by MR analysis and to determine if this relationship is bidirectional, spanning both men and women populations. By understanding these causal links, we aim to provide insights into how modulating gut microbiota can influence IGF-1 levels and vice versa, potentially offering new therapeutic targets for related diseases.

## Methods

### Data sources of gut microbiome

**Figure 1:**
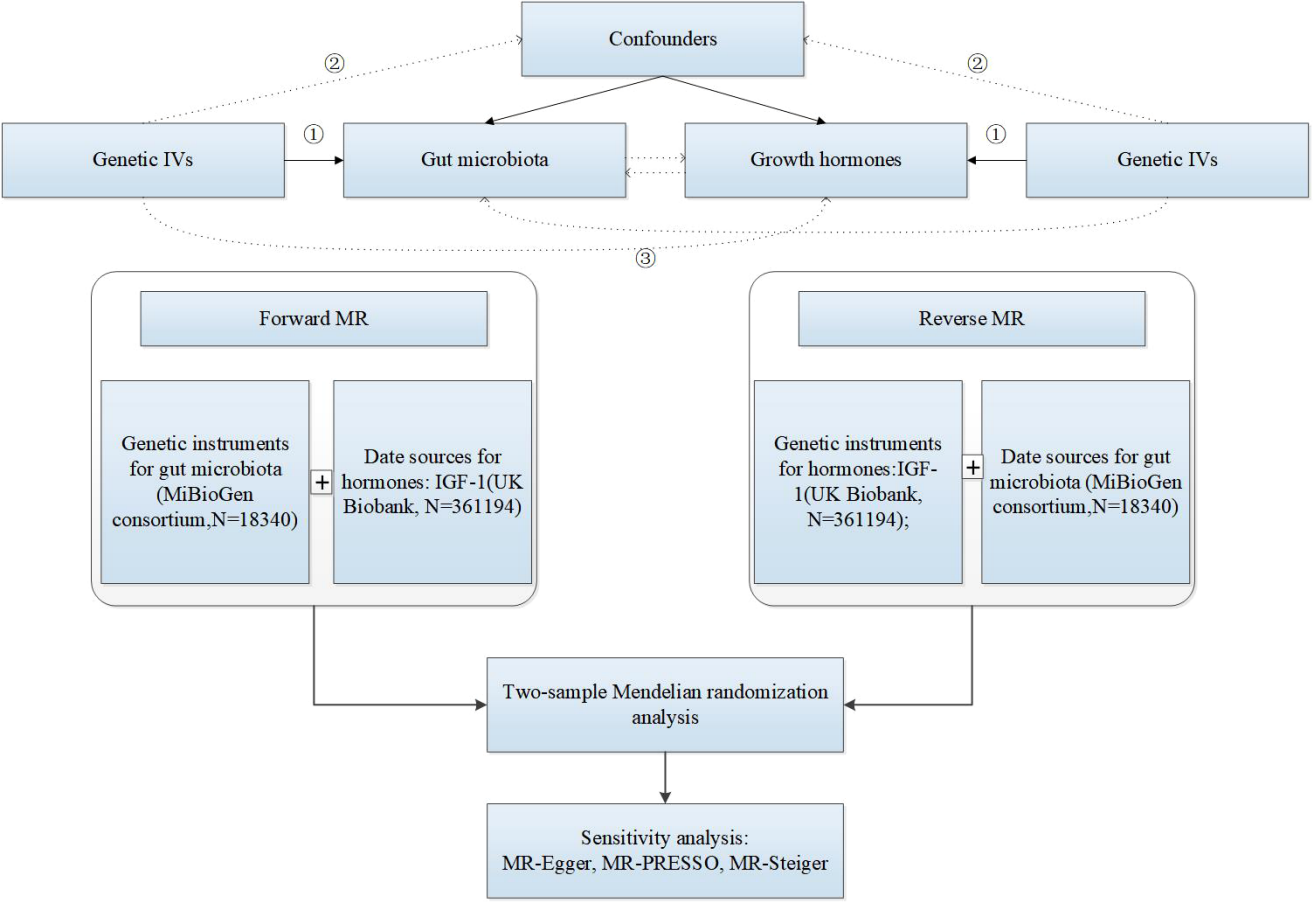
The research design and flow chart of MR. ① Genetic IVs are associated with exposure. ② Genetic IVs are not associated with confounders. ③ Genetic IVs influence outcome only through exposure.

The causal relationship between gut microbiota and IGF-1 was assessed by a bidirectional two-sample MR, and the study design and flowchart were shown in **Figure 1**. The MiBioGen consortium published a large-scale genome-wide association study (GWAS) of the composition of the gut microbiota (Kurilshikov et al., 2021). This dataset contained a total of 18,340 samples of 16S ribosomal ribonucleic acid (rRNA) gene sequencing data from 24 population-based cohorts. A total of 211 gut microbiomes were identified from genus to phylum. Subsequently, 15 bacteria were excluded due to unknown traits, and finally 119 genera, 32 families, 20 orders, 16 classes, and 9 phyla were included in the MR analysis. The original article described more detailed information about the gut microbiome (Yurkovetskiy et al., 2013).

### Data sources of IGF-1

Sex-specific datasets on IGF-1 were derived from the UK Biobank (http://www.nealelab.is/uk-biobank), utilizing GWAS summary statistics involving 361,194 participants of European ancestry. IGF-1 was measured using a chemiluminescent immunoassay (CLIA) (Siemens ADVIA Centaur IGF-1 assay). This assay is widely validated for accurate and reproducible measurement of IGF-1 levels across large populations. In the UK Biobank cohort, the reference range for serum IGF-1 levels varies by age and sex. For adults aged 18–35 years, the typical range is 116–358 ng/mL for men and 97–310 ng/mL for women. Principal component analysis was performed on the genetic data to adjust for population stratification, and the top 20 principal components were included as covariates in the analysis. These principal components capture the major axes of genetic variation within the dataset, thus controlling for potential confounding due to population structure. Genetic associations were also adjusted for age and age squared (age²), expressed as per standard deviation changes in IGF-1.

### Ethical approvement

All summary-level datasets in our study were retracted from de-identified public data/studies. Ethical approval and informed consent were obtained by the ethics committee previously. Ethical approval was thus exempted from our study.

### Genetic instrument selection

In this study, we followed strict criteria to ensure the robustness and validity of this MR study. For the gut microbiota as exposure, in order to obtain sufficient SNPs to be used as IVs, a *P*-value of <1e−5 was set as the significance threshold to select genetic instruments associated with bacterial traits. This threshold ensured that the selected SNPs are strongly associated with the exposure, reducing the risk of weak instrument bias. We set the chain imbalance threshold r^2^ to <0.001 and the distance to 10,000 kb to avoid this phenomenon of linkage disequilibrium. This process minimized the inclusion of correlated SNPs that might confound the MR analysis. IVs of the gut microbiota were shown in **Supplementary Table S1**. In addition, we used the MR Pleiotropy RESidual Sum and Outlier (MR-PRESSO) method to look for significant SNPs with pleiotropy (Verbanck et al., 2018) and excluded outliers if present. MR-PRESSO identified and removed significant outliers that might introduce pleiotropy, ensuring that the causal estimates were not biased by pleiotropic effects. The results of the F-statistic represent the strength of the IVs (F-statistics = Beta^2^/Se^2^, beta is the correlation coefficient between SNPs and traits) (GBD 2016 Headache Collaborators, 2018), and SNPs with F-statistic values > 10 indicate that there is no substantial weak instrumental bias; otherwise, the IVs were removed (Chen et al., 2022). Strong instruments were crucial for reliable causal inference in MR studies. For our study, all F-statistics were greater than 10. The participants in our analysis were derived from large-scale GWASs conducted by the MiBioGen consortium and the UK Biobank. The selection of participants in these studies is population-based, capturing a wide range of demographic and clinical characteristics. Therefore, the inclusion criteria primarily involve ensuring high-quality genotyping data and appropriate population structure.

### Statistical analysis

MR analysis must meet the following three assumptions to be performed correctly: (1) the assumption of relevance: the IVs used for the analysis should be closely related to the exposure; (2) the assumption of independence: the IVs were not related to the exposure or confounders of the outcome; and (3) the assumption of exclusivity: the IVs were not related to the outcome.

We used the inverse variance weighted (IVW) approach as the primary method to explore the causal relationship between exposure and outcome, an analysis that has the advantage of providing robust causality estimates in the absence of directional pleiotropy (consistent with the independence assumption) (Ehret et al., 2011). In addition, three other additional analytical methods, namely, MR-Egger, weighted median (WM) analysis, and weighted mode method, were conducted as secondary references to improve the reliability of causality. The MR-Egger method was based on the assumption that all IVs are invalid and the intercept term is present by default (Bowden et al., 2015). The WM analysis method was based on the assumption that more than 50% of SNPs have valid SNPs (Bowden et al., 2016). For estimation methods based on weighted models, smaller sample sizes are required, but lower type I error rates and smaller biases are guaranteed. *P* < 0.05 indicates statistical significance. We also adjusted for the results in multiple comparisons (Benjamini and Hochberg) by false discovery rate (FDR). All MR analyses in this study were performed in the R software (version 4.2.0, The R Foundation, Vienna, Austria) with the “TwoSampleMR,” “MR-PRESSO,” “frostplot,” and “ggplot2” packages.

### Sensitivity analysis

The purpose of the sensitivity analysis was to test for heterogeneity and horizontal pleiotropy of IVs in MR analysis. We performed Cochran’s Q test for heterogeneity of IVs with MR-Egger and IVW methods. Heterogeneity could indicate that the instruments were not consistently estimating the same causal effect, which could bias the results. Cochran’s Q test compared the observed variance among the effect estimates of the genetic instruments to what would be expected if all instruments were estimating the same effect. A P-value greater than 0.05 from Cochran’s Q test indicated that there was no significant heterogeneity among the IVs, suggesting that the instruments are homogeneous and the causal estimates are reliable. In addition, horizontal pleiotropy was assessed using two methods: MR Egger intercept and MR-PRESSO global test. The MR Egger regression method provides an intercept term that can be used to test for directional pleiotropy. A significant non-zero intercept indicates the presence of pleiotropy, which can bias the MR estimates. In our analysis, a p-value greater than 0.05 for the intercept term suggests that there is no evidence of horizontal pleiotropy, indicating that the genetic instruments are not affecting the outcome through pathways other than the exposure. MR-PRESSO global test method is used to detect and correct for horizontal pleiotropy. This method identifies significant outliers that may contribute to pleiotropy. The global test within MR-PRESSO assesses the overall pleiotropy by comparing the observed data to the expected distribution under no pleiotropy. A p-value greater than 0.05 indicates the absence of significant pleiotropy. Furthermore, MR-PRESSO analysis reveals specific SNP outliers that contribute to pleiotropy, which can be removed to refine the causal estimates and reduce bias. To further validate the stability of our results, we performed a “leave-one-out” analysis, which evaluates the influence of each individual SNP on the overall causal estimate. Of course, we used PhenoScanner (BMI and smoking status) to exclude potentially pleiotropic SNPs that were significantly associated with confounding factors (http://www.phenoscanner.medschl.cam.ac.uk/).

### Reverse MR analysis of the causal effects of IGF-1 on gut microbiome

For the reverse MR analysis, we collected IVs at a threshold of P < 1e-8 (**Supplementary Tables S2**, **S3**) in order to explore the genetically predicted causal effects of IGF-1 on the gut microbiota. The chain imbalance threshold r^2^ was also set to < 0.001, and the distance is set to 10,000 kb. We had previously described the statistical methods to be used in the reverse MR analysis. Our study was conducted in accordance with the STROBE-MR checklist (**STROBE-MR-checklist**) (Skrivankova et al., 2021).

## Results

### Causal effects of the gut microbiome on IGF-1 in MR analysis

**Figure 2:**
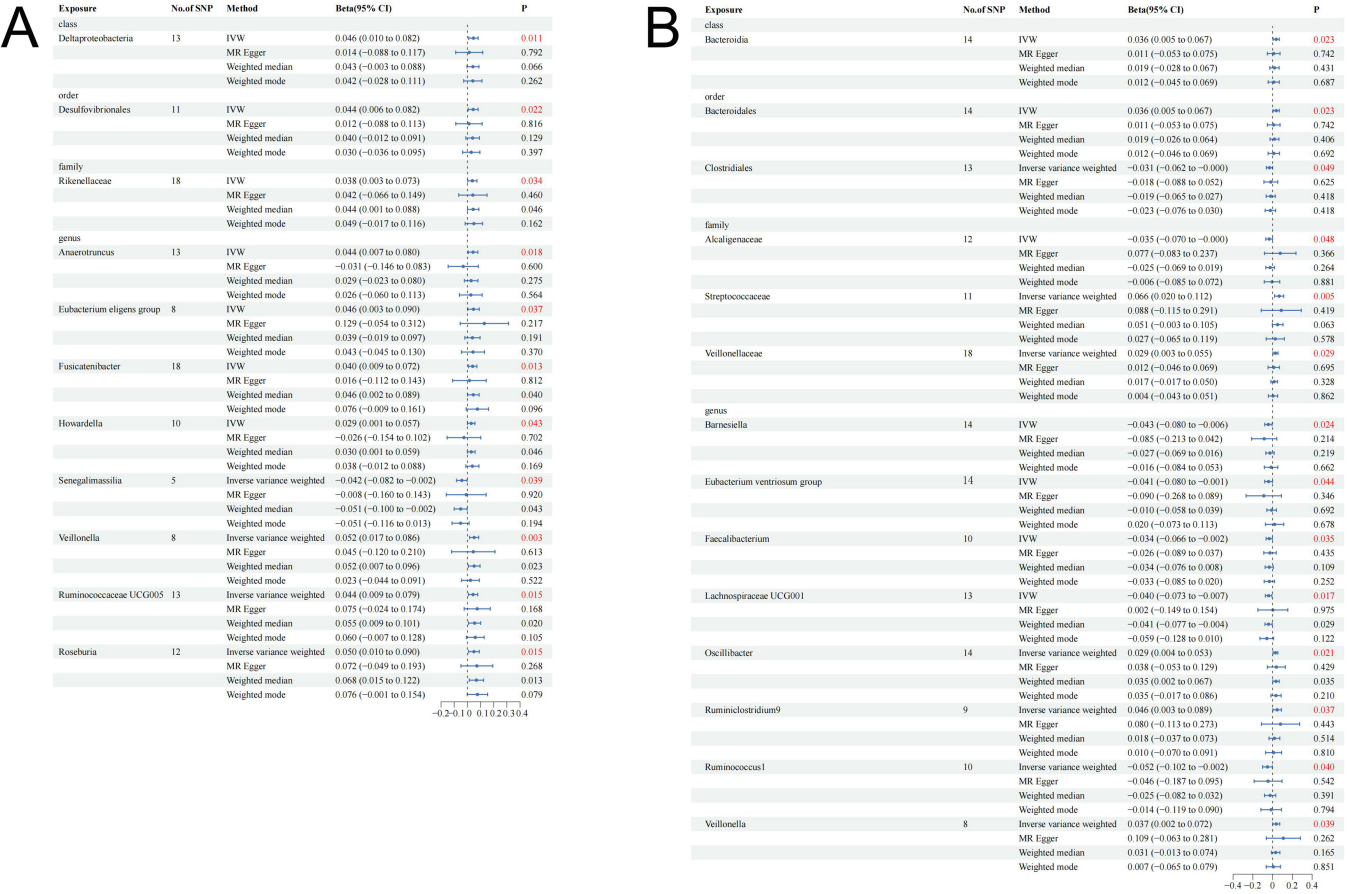
Causal effect estimates of gut microbiota on IGF-1 in men and women. **(A)** Men. **(B)** Women. CI, confidence interval; IVW, inverse variance weighted method.

The research design and flow chart of this study was shown in **Figure 1**. A total of 196 bacterial traits from five biological levels (phylum, order, family, and genus) were finally included in this study. In the forward MR analysis, the causal effects of 196 bacterial taxa on IGF-1 in men and women were shown in **Supplementary Figures S1**, **S2**, respectively. As shown in **Table 1**, genetically predicted class Deltaproteobacteria [beta = 0.046, 95% confidence interval (CI) = 0.010 to 0.082, *P* = 0.011], order Desulfovibrionales (beta = 0.044, 95% CI = 0.006 to 0.082*, P =* 0.022), family Rikenellaceae (beta = 0.038, 95% CI = 0.003 to 0.073, *P =* 0.034), genus *Anaerotruncus* (beta = 0.044, 95% CI = 0.007 to 0.080, *P =* 0.018), genus *Eubacterium eligens* group (beta = 0.046, 95% CI = 0.003 to 0.090, *P =* 0.037), genus *Fusicatenibacter* (beta = 0.040, 95% CI = 0.009 to 0.072, *P =* 0.013), genus *Howardella* (beta = 0.029, 95% CI = 0.001 to 0.057, *P =* 0.043), genus *Senegalimassilia* (beta = −0.042, 95% CI = −0.082 to −0.002, *P =* 0.039), genus *Veillonella* (beta = 0.052, 95% CI = 0.017 to 0.086, *P =* 0.003), genus *Ruminococcaceae* UCG005 (beta = 0.044, 95% CI = 0.009 to 0.079, *P =* 0.015), and genus *Roseburia* (beta = 0.050, 95% CI = 0.010 to 0.090, *P =* 0.015) had a causal effect on IGF-1 in men (**Figure 2A**), whereas genetically predicted class Bacteroidia (beta = 0.036, 95% CI = 0.005 to 0.067, *P* = 0.023), order Bacteroidales (beta = 0.036, 95% CI = 0.005 to 0.067, *P =* 0.023), order Clostridiales (beta = −0.031, 95% CI = −0.062 to −0.000, *P =* 0.049), family Alcaligenaceae (beta = −0.035, 95% CI = −0.070 to −0.000, *P =* 0.048), family Streptococcaceae (beta = −0.066, 95% CI = 0.020 to 0.112, *P =* 0.005), family Veillonellaceae (beta = −0.029, 95% CI = 0.003 to 0.055, *P =* 0.029), genus *Barnesiella* (beta = −0.043, 95% CI = −0.080 to −0.006, *P =* 0.024), genus *Eubacterium ventriosum* group (beta = −0.041, 95% CI = −0.080 to −0.001, *P =* 0.044), genus *Faecalibacterium* (beta = −0.034, 95% CI = −0.066 to −0.002, *P =* 0.035), genus *Lachnospiraceae* UCG001 (beta = −0.040, 95% CI = −0.073 to −0.007, *P =* 0.017), genus *Oscillibacter* (beta = 0.029, 95% CI = 0.004 to 0.053, *P =* 0.021), genus *Ruminiclostridium9* (beta = 0.046, 95% CI = 0.003 to 0.089, *P =* 0.037), genus *Ruminococcus1* (beta = −0.052, 95% CI = −0.102 to −0.002, *P =* 0.040), and genus *Veillonella* (beta = 0.037, 95% CI = 0.002 to 0.072, *P =* 0.039) had a causal effect on IGF-1 in women (**Figure 2B**). Overall, our forward MR analysis identified 11 bacterial taxa with a causal effect on IGF-1 in men and 14 in women. Compared to observational studies, our study provides stronger causal inferences.

**Table 1:** Positive MR results for the causal impact of gut microbiota on IGF-1 in men and women.

Sex	Exposure	No. SNP	F-statistics	Method	Beta (95% CI)	Pval
**Men**	**Class**					
	Deltaproteobacteria	13	21.06	IVW	0.046 (0.010 to 0.082)	0.011
	**Order**					
	Desulfovibrionales	11	21.33	IVW	0.044 (0.006 to 0.082)	0.022
	Family					
	Rikenellaceae	18	21.61	IVW	0.038 (0.003 to 0.073)	0.034
	**Genus**					
	Anaerotruncus	13	20.84	IVW	0.044 (0.007 to 0.080)	0.018
	Eubacterium eligens group	8	20.69	IVW	0.046 (0.003 to 0.090)	0.037
	Fusicatenibacter	18	21.12	IVW	0.040 (0.009 to 0.072)	0.013
	Howardella	10	21.74	IVW	0.029 (0.001 to 0.057)	0.043
	Senegalimassilia	5	20.94	IVW	−0.042 (−0.082 to −0.002)	0.039
	Veillonella	8	20.97	IVW	0.052 (0.017 to 0.086)	0.003
	Ruminococcaceae UCG005	13	21.21	IVW	0.044 (0.009 to 0.079)	0.015
	Roseburia	12	21.43	IVW	0.050 (0.010 to 0.090)	0.015
**Women**	**Class**					
	Bacteroidia	14	21.09	IVW	0.036 (0.005 to 0.067)	0.023
	**Order**
	Bacteroidales	14	21.09	IVW	0.036 (0.005 to 0.067)	0.023
	Clostridiales	13	21.73	IVW	−0.031 (−0.062 to −0.000)	0.049
	**Family**					
	Alcaligenaceae	12	21.94	IVW	−0.035 (−0.070 to −0.000)	0.048
	Streptococcaceae	11	22.58	IVW	0.066 (0.020 to 0.112)	0.005
	Veillonellaceae	18	21.23	IVW	0.029 (0.003 to 0.055)	0.029
	**Genus**					
	*Barnesiella*	14	21.94	IVW	−0.043 (−0.080 to −0.006)	0.024
	Eubacterium ventriosum group	14	21.36	IVW	−0.041 (−0.080 to −0.001)	0.044
	Faecalibacterium	10	21.09	IVW	−0.034 (−0.066 to −0.002)	0.035
	Lachnospiraceae UCG001	13	22.08	IVW	−0.040 (−0.073 to −0.007)	0.017
	Oscillibacter	14	22.21	IVW	0.029 (0.004 to 0.053)	0.021
	Ruminiclostridium9	9	21.07	IVW	0.046 (0.003 to 0.089)	0.037
	Ruminococcus1	10	21.05	IVW	−0.052 (−0.102 to −0.002)	0.040
	Veillonella	8	20.97	IVW	0.037 (0.002 to 0.072)	0.039

MR, Mendelian randomization; IGF-1, insulin-like growth factor 1; No. SNP, number of SNPs; CI, confidence interval.

In the forward MR analysis, all bacterial traits failed to pass the FDR correction (*P* > 0.05). In the sensitivity analysis, Cochran’s Q test showed no sign of heterogeneity for all bacterial traits (**Supplementary Table S4**). No pleiotropy was found in the results by MR-Egger and MR-PRESSO analytical methods (**Supplementary Table S4**). In addition, the results of leave-one-out analysis provide further evidence of the robustness of the results (**Supplementary Figures S3**, **S4**). The power results showed that the power to evaluate the causal effects of these microbiota features and IGF-1 was satisfied; most of them were >70%.

### Causal effects of the genetically predicted IGF-1 on gut microbiome in reverse MR analysis

**Figure 3:**
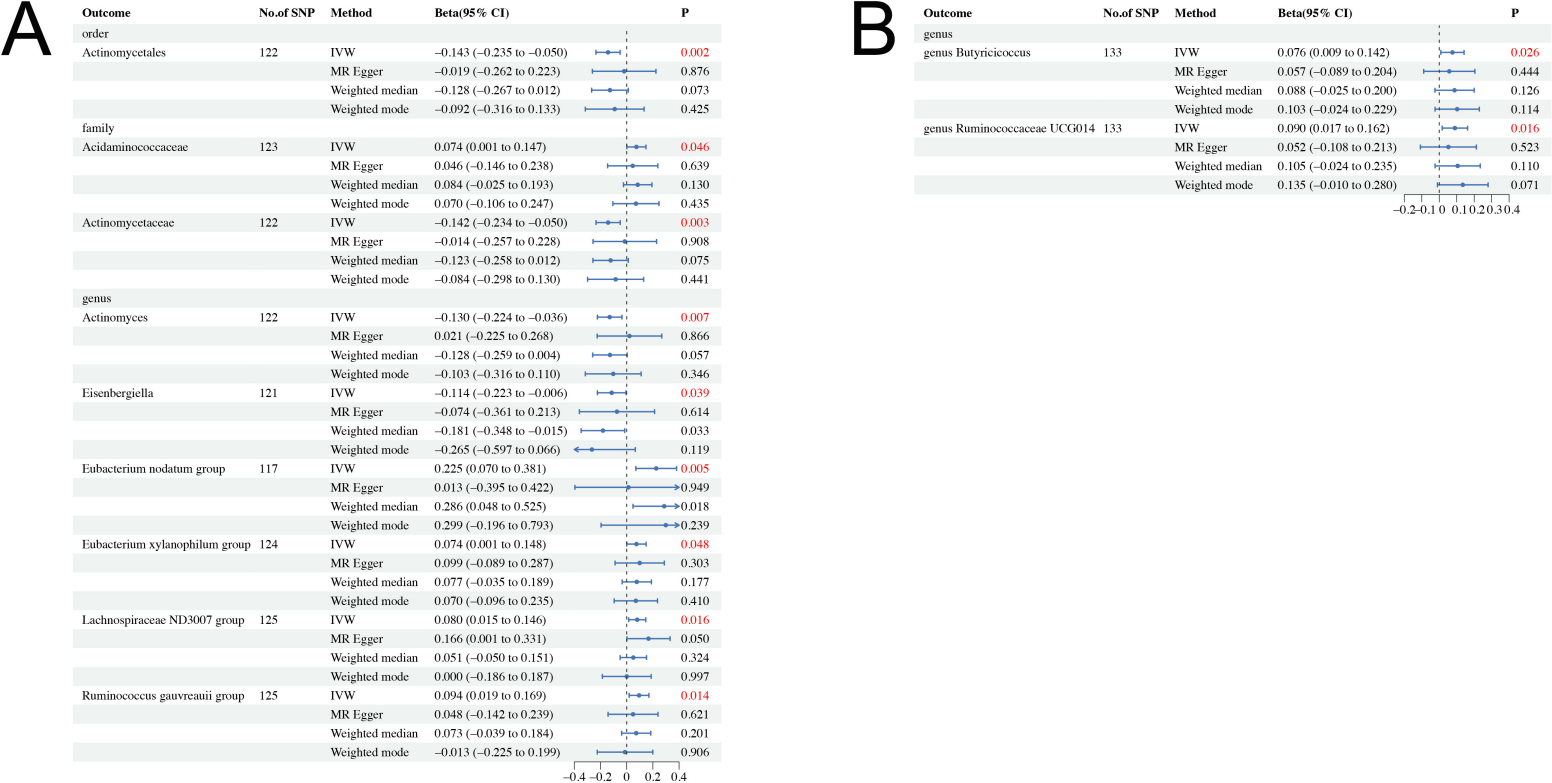
Causal effect estimates of IGF-1 on gut microbiota in men and women. **(A)** Men. **(B)** Women. CI, confidence interval; IVW, inverse variance weighted method.

The causal effects of total IGF-1 on 196 bacterial taxa in reverse MR analysis for men and women were shown in **Supplementary Figures S5**, **S6**, respectively. As shown in **Table 2**, IGF-1 levels increased bacterial abundance of family Acidaminococcaceae (beta = 0.074, 95% CI = 0.001 to 0.147, *P =* 0.046), genus *Eubacterium nodatum* group (beta = 0.025, 95% CI = 0.070 to 0.381, *P =* 0.005), genus *Eubacterium xylanophilum* group (beta = 0.074, 95% CI = 0.001 to 0.148, *P =* 0.048), genus *Lachnospiraceae* ND3007 group (beta = 0.080, 95% CI = 0.015 to 0.146, *P =* 0.016), and genus *Ruminococcus gauvreauii* group (beta = 0.094, 95% CI = 0.019 to 0.169, *P =* 0.014) in men. In contrast, the bacterial abundance of family Actinomycetaceae (beta = −0.142, 95% CI = −0.234 to −0.050, *P =* 0.003), genus *Actinomyces* (beta = −0.130, 95% CI = −0.224 to −0.036, *P =* 0.007), and genus *Eisenbergiella* (beta = −0.114, 95% CI = −0.223 to −0.006, *P =* 0.039) were decreased in men (**Figure 3A**). Moreover, genetically predicted IGF-1 levels increased bacterial abundance of genus *Butyricicoccus* (beta = 0.076, 95% CI = 0.009 to 0.142, *P =* 0.026), and genus *Ruminococcaceae* UCG014 (beta = 0.090, 95% CI = 0.017 to 0.162, *P =* 0.016) in women (**Figure 3B**). Therefore, the results of our reverse MR analysis indicated that IGF-1 exerted a causal effect on nine bacterial taxa in males and two bacterial taxa in females, respectively.

**Table 2:** Positive MR results for the causal impact of IGF-1 on gut microbiota in men and women.

Sex	No. SNP	F-statistics	Outcome	Method	Beta (95% CI)	Pval
**Men**			**Order**			
	122	80.38	Actinomycetales	IVW	−0.143 (−0.235 to −0.050)	0.002
			**Family**			
	123	80.33	Acidaminococcaceae	IVW	0.074 (0.001 to 0.147)	0.046
	122	80.38	Actinomycetaceae	IVW	−0.142 (−0.234 to −0.050)	0.003
			**Genus**			
	122	80.38	Actinomyces	IVW	−0.130 (−0.224 to −0.036)	0.007
	121	80.80	Eisenbergiella	IVW	−0.114 (−0.223 to −0.006)	0.039
	117	81.96	Eubacterium nodatum group	IVW	0.225 (0.070 to 0.381)	0.005
	124	85.06	Eubacterium xylanophilum group	IVW	0.074 (0.001 to 0.148)	0.048
	125	84.99	Lachnospiraceae ND3007 group	IVW	0.080 (0.015 to 0.146)	0.016
	125	84.99	Ruminococcus gauvreauii group	IVW	0.094 (0.019 to 0.169)	0.014
**Women**			**Genus**			
	133	80.31	Butyricicoccus	IVW	0.076 (0.009 to 0.142)	0.026
	133	80.31	Ruminococcaceae UCG014	IVW	0.090 (0.017 to 0.162)	0.016

MR, Mendelian randomization; IGF-1, insulin-like growth factor 1; No. SNP, number of SNPs; CI, confidence interval.

**Figure 4:**
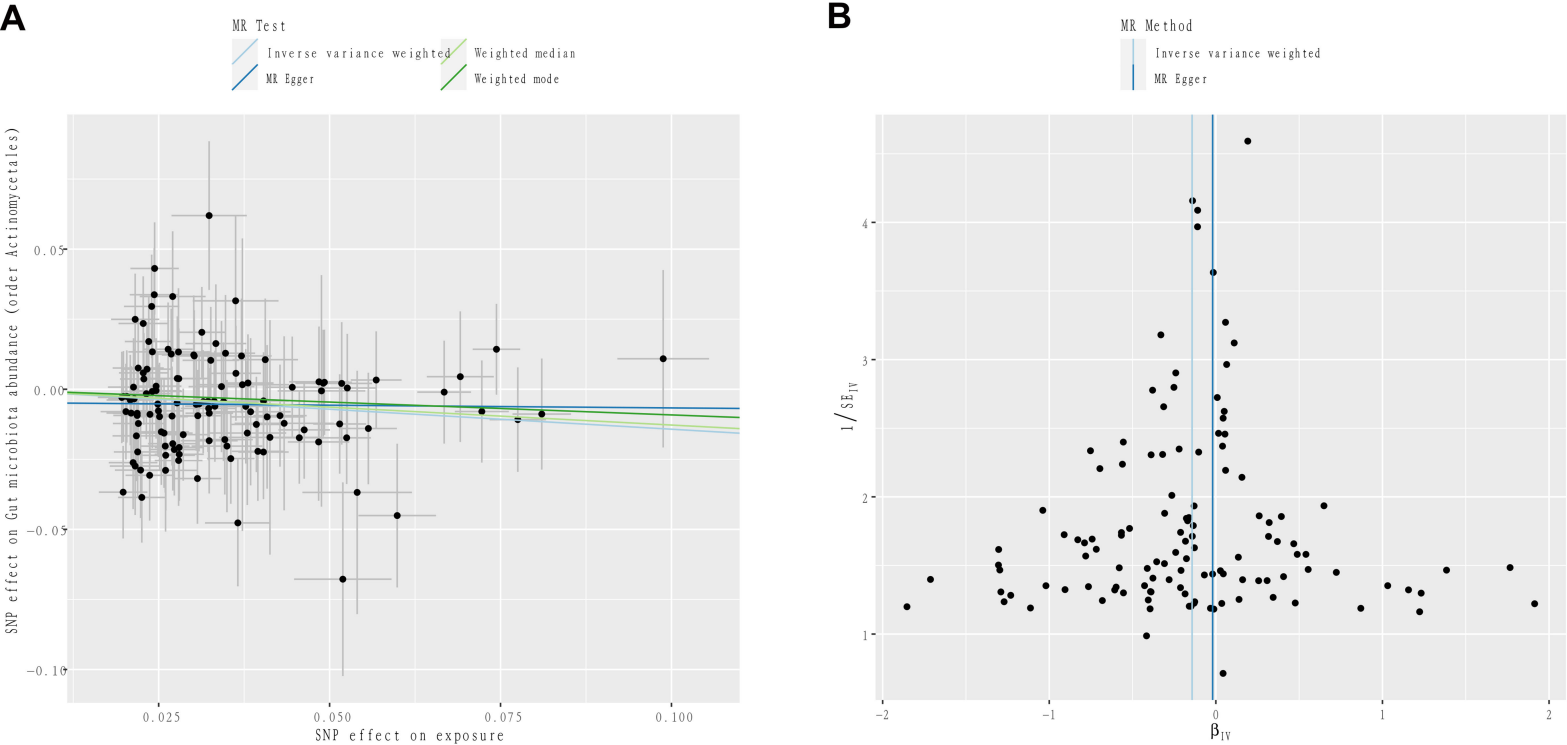
**(A)** Scatter plots to visualize the causal effect of IGF-1 on order Actinomycetales. **(B)** Funnel plots to visualize overall heterogeneity of MR estimates for the effect of IGF-1.

After FDR correction, the causal effect of IGF-1 on order Actinomycetales (*P_FDR_* =0.049) remained in men. **Figure 4** further demonstrates the stability of this result. In the sensitivity analysis, the results of Cochran’s Q test showed no signs of heterogeneity for all bacterial traits (**Supplementary Table S5**). No pleiotropy was found in the results by MR-Egger and MR-PRESSO analytical methods (**Supplementary Table S5**). In addition, the results of leave-one-out analysis provided further evidence of the robustness of the results (**Supplementary Tables S6**, **S7**).

In summary, the results indicated that specific bacterial taxa were closely linked with IGF-1 levels, suggesting potential pathways through which gut microbiota might influence endocrine function.

## Discussion

In this MR study, this study is the first to investigate the causal relationship between gut microbiota IGF-1 through large-scale GWAS summary data. Our findings provide new insights into the complex interplay between gut microbiota and growth factors, which have significant implications for understanding metabolic and endocrine disorders.

IGF-1, as a growth factor, has been shown to have a bidirectional causal relationship with the gut microbiota. First, how the microbiota affects systemic and localized IGF-1 is still under investigation. IGF-1 is downstream of growth hormone during postnatal development (Van Wyk and Smith, 1999), and whether the microbiota mediates IGF-1 production via growth hormone is unknown. There were substantial differences in IGF-1 levels but similar levels of circulating growth hormone in conventionally reared mice, colonized mice, and germ-free mice (Schwarzer et al., 2016; Yan et al., 2016). Similarly, the pathway by which *E. coli* colonization regulates IGF-1 levels is independent of growth hormone because serum growth hormone in *B. thailandensis–*infected mice is not altered after *E. coli* O21:H+ colonization. In summary, IGF-1 production and function are not only mediated by growth hormone but also altered with changes in the gut microbiota. Our study corroborates these findings and further elucidates the directionality of these relationships. We demonstrated that certain bacterial taxa causally influence IGF-1 levels and vice versa. Specifically, our forward MR analysis identified 11 bacterial taxa with a causal effect on IGF-1 in men and 14 in women. Compared to observational studies, our study provides stronger causal inferences.

Many different bacterial species can regulate IGF-1 levels, so the common production of a microbial metabolite by these species, short-chain fatty acids (SCFAs), has the potential to provide an explanation for the mechanism by which the gut microbiota regulates host IGF-1. SCFAs, including acetate, propionate, and butyrate, represent a wide range of microbial metabolites generated through the fermentation of non-digestible dietary fibers. Among these, butyrate stands out as the primary source of energy for enterocytes. SCFA can exert their effects both within the local environment of the intestinal tract and systemically by entering the bloodstream (Yan and Charles, 2018). SCFA concentrations in the feces of conventionally reared mice were higher than those in germ-free mice (Arpaia et al., 2013; Smith et al., 2013). In a study by Yan et al (Yan et al., 2016), conventional mice treated with broad-spectrum antibiotics and vancomycin experienced a reduction in SCFA concentrations, whereas the cecum of colonized germ-free mice exhibited increased SCFA levels. Additionally, as cecal SCFA concentrations roughly correlated with trends in serum IGF-1, they demonstrated that, akin to colonization in mice, supplementation with SCFA led to enhanced production of IGF-1 in adipose tissue and a noticeable trend toward increased IGF-1 production in the liver (Yan et al., 2016). This suggests that the gut microbiota may influence IGF-1 production either directly or indirectly through the generation of SCFA. A comprehensive review supports the link between microbially produced SCFA and IGF-1, reporting that feeding non-digestible fiber, oligosaccharides (fermented into SCFA), and probiotics promotes bone health (McCabe et al., 2015). However, it is not possible to definitively conclude that SCFA were sufficient to directly induce IGF-1, and it was likely that additional microbiota–host interactions contribute to the increased IGF-1 production by host tissues. SCFA may indirectly influence the production of IGF-1 through the following mechanisms: SCFA can improve insulin sensitivity, thereby enhancing the anabolic effects of insulin and subsequently influencing the production of IGF-1 (Canfora et al., 2015); SCFA can strengthen gut barrier integrity, supporting the nutrient absorption necessary for IGF-1 synthesis (Koh et al., 2016); SCFA can regulate lipid metabolism, maintaining lipid balance, which is important for metabolic health and can indirectly affect IGF-1 (den Besten et al., 2013).

One of the pioneering studies to investigate the impact of IGF-1 on the gut microbiota involved its administration to nutritionally restricted female BALB/c mice (Chen et al., 2016). Initially, the gut microbiota in the nutritionally restricted mice showed signs of dysregulation and immaturity. Subsequently, a subset of the diet-restricted BALB/c mice underwent treatment with food restoration alone or a combination of food restoration along with subcutaneous IGF-1 injections. The results revealed that IGF-1 not only restored body weight but also mitigated dysbiosis and immaturity in the gut microbiota, ultimately bringing it back to a composition similar to the *ad libitum* group, independent of diet (Chen et al., 2016). Additionally, Buford et al. identified a positive correlation between IGF-1 levels and certain taxonomic groups, including the Leptospirae family, Bacteroidetes spp. (synonym Bacteroidota), TM7, and Tenericutes phyla (Buford et al., 2018). Another study demonstrated that the specific deletion of IGF-1 in the intestinal epithelial cells of mice (conditional knockout (cKO) mice) induced changes in the microbial composition of the cecum, leading to a decrease in *Helicobacter* spp., *Lactobacillus* spp., and *Oscillospira* spp., as well as an increase in Odoribacte and Bacteroides (Zheng et al., 2018). On this basis, the results of our reverse MR analysis demonstrated that IGF-1 has a causal effect on nine bacterial taxa in men and two bacterial taxa in women, respectively. After FDR correction, the causal effect of IGF-1 on order Actinomycetales (P_FDR_=0.049) remained in men.

IGF-1 exerts various biological functions in the body and may influence the composition and function of the gut microbiota through multiple mechanisms. Firstly, IGF-1 can affect the gut microbiota by regulating the host immune system (Duncan et al., 1994). An intact gut barrier helps prevent pathogen invasion while supporting the colonization of beneficial microbes. Additionally, IGF-1 can modulate the function of immune cells such as macrophages and T cells, which, in turn, influences the microbial environment in the gut (Spadaro et al., 2017). Secondly, IGF-1 indirectly regulates the gut microbiota by affecting the secretory activities of intestinal epithelial cells, such as mucus and antimicrobial peptide production (Sheng et al., 2011). These secretions can selectively inhibit the growth of harmful bacteria while promoting the proliferation of beneficial microbes, thus altering the composition of the gut microbiota. Lastly, IGF-1 can influence the gut microbiota through interactions with metabolites. IGF-1 is involved in regulating the body’s metabolic processes, including glucose and lipid metabolism (Yakar et al., 2002). These metabolites can serve as nutrients or signaling molecules for microbes, thereby affecting the growth and metabolic activities of the gut microbiota.

While our study primarily focused on the bacterial component of the gut microbiome, we acknowledge that these other microbial components could also play significant roles. Gut archaea, such as methanogens, play a role in maintaining gut homeostasis by participating in the digestion of complex carbohydrates and the production of methane. This metabolic activity can influence the overall gut environment, potentially affecting the composition and function of bacterial communities and their interactions with IGF-1 levels (Samuel and Gordon, 2006). Bacteriophages can modulate bacterial populations by infecting and lysing specific bacterial hosts. This dynamic interaction can lead to shifts in the bacterial community structure, indirectly influencing metabolic functions and the production of metabolites like SCFAs, which are known to affect IGF-1 levels (Duerkop and Hooper, 2013). Gut fungi, although present in smaller numbers compared to bacteria, can interact with bacterial communities and the host immune system. Fungi can influence inflammatory responses and nutrient metabolism, which could indirectly impact IGF-1 production (Mukherjee et al., 2015). The oral microbiome can contribute to systemic inflammation and metabolic changes through the translocation of oral bacteria into the gut and bloodstream. This process can affect gut microbial composition and metabolic pathways, potentially influencing IGF-1 levels (Atarashi et al., 2011). Due to data limitations, we were unable to explore the specific influences of gut archaea, viruses, fungi, and the oral microbiome on IGF-1 levels in this analysis. However, their potential contributions should be considered in the interpretation of our findings.

The bidirectional causal relationship between gut microbiota and IGF-1 has important implications for understanding the underlying mechanisms of metabolic and endocrine disorders. For instance, the identified causal effects of gut microbiota on IGF-1 levels suggest potential therapeutic targets for modulating IGF-1 levels through microbiota interventions. Conversely, understanding how IGF-1 influences gut microbiota composition could lead to novel strategies for managing conditions such as obesity, diabetes, and growth disorders. Our findings also underscore the importance of considering both directions of causality in future research to fully capture the complexity of these interactions.

This study, while offering valuable insights, is not without its limitations. Firstly, it is important to note that the genetic data pooled from GWAS primarily included European participants, which may restrict the applicability of our findings to other populations, thereby constraining the generalizability of our results. Secondly, our analytical scope was constrained by the capabilities of our classifiers and sequencing methods, which permitted us to examine the gut microbiota only at the genus level and higher taxonomic classifications. Thirdly, the inherent limitations of the available data prevented us from assessing individual-level associations. Additionally, although our MR approach provides robust causal inference, several potential confounders and biases, including population stratification, measurement error, and reverse causation, still need to be considered. Population stratification can introduce bias if there are systematic differences in allele frequencies between subpopulations with different ancestries. IGF-1 data source was adjusted for the top 20 principal components in the analysis, which capture the major axes of genetic variation and help control for population structure. Despite these adjustments, residual confounding due to population stratification may still exist and should be acknowledged. In our study, we used well-established GWAS data from large consortia (MiBioGen and UK Biobank), which have rigorous quality control measures to minimize measurement error. However, it is important to recognize that residual measurement error cannot be entirely ruled out, and such errors may attenuate the estimated causal effects. Our bidirectional MR approach helps mitigate this concern by examining the causal effects in both directions. Nonetheless, it is essential to interpret the findings in the context of potential reverse causation and consider additional evidence from experimental or longitudinal studies to strengthen the causal inference. Other important confounding factors, such as overall health status, nutritional status, and detailed dietary habits, could not be explored in our analysis due to data limitations. Lastly, the MR itself has limitations, as it does not take into account epigenetic modifications, which may affect gene expression and IGF-1 production. The MR assumes a direct causal pathway, oversimplifying the complex biological processes involved, such as the roles of bacterial metabolites, inflammation, and nutrient absorption. Additionally, although we used stringent criteria to select genetic instruments, residual pleiotropy could still bias our results. Further studies with larger sample sizes are needed to confirm these findings.

## Conclusion

Our study demonstrates a bidirectional causal link between the gut microbiota and IGF-1, spanning both men and women populations. Even after correction, most of the results became non-significant but could still suggest an association between gut microbiota and IGF-1. This discovery implies that the associated microbiota are potential therapeutic targets for promoting homeostasis of these hormones, which, in turn, may modulate gut microbiota homeostasis. Certainly, future studies need to validate the causal relationship between the gut microbiome and IGF-1 and delve into the intricate mechanisms that underlie this connection.

## Data Availability

The original contributions presented in the study are included in the article/**Supplementary Material**. Further inquiries can be directed to the corresponding author.

## Supplementary material

Supplementary Table 1 Single-nucleotide polymorphisms used as instrumental variables for gut microbiota at the threshold of P<1e-5 in MR analysis.

Supplementary Table 2 Single nucleotide polymorphisms used as instrumental variables for IGF-1 in men at the threshold of P<1e-8 in reverse MR analysis.

Supplementary Table 3 Single nucleotide polymorphisms used as instrumental variables for IGF-1 in women at the threshold of P<1e-8 in reverse MR analysis.

Supplementary Table 6 The leave-one-out analysis of IGF-1 on the gut microbiome in men.

Supplementary Table 7 The leave-one-out analysis of IGF-1 on the gut microbiome in women.

SUPPLEMENTARY DATA SHEET 1 Code and comments for MR analysis.

SUPPLEMENTARY DATA SHEET 2 STROBE-MR-checklist.
