# Supplementary material for: Causal relationship between gut microbiota and insulin-like growth factor 1: a bidirectional two-sample Mendelian randomization study
Source: Front Cell Infect Microbiol. 2024 Sep 24;14:1406132. doi: 10.3389/fcimb.2024.1406132 (PMC11463061; doi:10.3389/fcimb.2024.1406132)
Supplement: Supplementary Table 6 — The leave-one-out analysis of IGF-1 on the gut microbiome in men. [file Table2.docx]

**Table S4 Sensitivity analysis of significant gut microbiota on IGF-1 in men and women.**

| **Outcome** | **Sex** | **Exposure** | **Heterogeneity test** | | | Pleiotropy test | |
| --- | --- | --- | --- | --- | --- | --- | --- |
|  |  |  | **Method** | **Cochran’s Q** | **Cochran’s P** | Egger_intercept | P-Egger intercept |
| **IGF-1** | Men | **Class** |  |  |  |  |  |
|  |  | Deltaproteobacteria | MR-Egger | 13.36 | 0.270 | 0.0025 | 0.525 |
|  |  |  | IVW | 13.89 | 0.308 |  |  |
|  |  | **Order** |  |  |  |  |  |
|  |  | Desulfovibrionales | MR-Egger | 10.84 | 0.287 | 0.0025 | 0.517 |
|  |  |  | IVW | 10.39 | 0.328 |  |  |
|  |  | **Family** |  |  |  |  |  |
|  |  | Rikenellaceae | MR-Egger | 21.66 | 0.155 | -0.0003 | 0.941 |
|  |  |  | IVW | 21.67 | 0.198 |  |  |
|  |  | **Genus** |  |  |  |  |  |
|  |  | Anaerotruncus | MR-Egger | 6.09 | 0.868 | 0.0052 | 0.203 |
|  |  |  | IVW | 7.92 | 0.791 |  |  |
|  |  | Eubacterium eligens group | MR-Egger | 4.17 | 0.654 | -0.0068 | 0.399 |
|  |  |  | IVW | 4.99 | 0.661 |  |  |
|  |  | Fusicatenibacter | MR-Egger | 11.40 | 0.784 | 0.0017 | 0.701 |
|  |  |  | IVW | 11.56 | 0.826 |  |  |
|  |  | Howardella | MR-Egger | 16.07 | 0.041 | 0.0082 | 0.412 |
|  |  |  | IVW | 17.58 | 0.040 |  |  |
|  |  | Senegalimassilia | MR-Egger | 4.19 | 0.242 | -0.0033 | 0.680 |
|  |  |  | IVW | 4.48 | 0.345 |  |  |
|  |  | Veillonella | MR-Egger | 3.40 | 0.757 | 0.0006 | 0.937 |
|  |  |  | IVW | 3.41 | 0.845 |  |  |
|  |  | Ruminococcaceae UCG005 | MR-Egger | 13.05 | 0.290 | -0.0028 | 0.522 |
|  |  |  | IVW | 13.57 | 0.329 |  |  |
|  |  | Roseburia | MR-Egger | 12.34 | 0.263 | -0.0018 | 0.710 |
|  |  |  | IVW | 12.53 | 0.325 |  |  |
|  | Women | **Class** |  |  |  |  |  |
|  |  | Bacteroidia | MR-Egger | 9.64 | 0.723 | 0.0022 | 0.396 |
|  |  |  | IVW | 7.27 | 0.700 |  |  |
|  |  | **Order** |  |  |  |  |  |
|  |  | Bacteroidales | MR-Egger | 8.86 | 0.715 | 0.0022 | 0.396 |
|  |  |  | IVW | 9.64 | 0.723 |  |  |
|  |  | Clostridiales | MR-Egger | 11.33 | 0.416 | -0.0011 | 0.685 |
|  |  |  | IVW | 11.51 | 0.486 |  |  |
|  |  | **Family** |  |  |  |  |  |
|  |  | Alcaligenaceae | MR-Egger | 6.32 | 0.400 | -0.0073 | 0.189 |
|  |  |  | IVW | 8.31 | 0.788 |  |  |
|  |  | Streptococcaceae | MR-Egger | 16.95 | 0.051 | -0.0018 | 0.835 |
|  |  |  | IVW | 17.04 | 0.074 |  |  |
|  |  | Veillonellaceae | MR-Egger | 18.88 | 0.275 | 0.0015 | 0.515 |
|  |  |  | IVW | 19.41 | 0.306 |  |  |
|  |  | **Genus** |  |  |  |  |  |
|  |  | Barnesiella | MR-Egger | 20.96 | 0.051 | 0.0036 | 0.505 |
|  |  |  | IVW | 21.78 | 0.059 |  |  |
|  |  | Eubacterium ventriosum group | MR-Egger | 20.23 | 0.063 | 0.0037 | 0.592 |
|  |  |  | IVW | 20.74 | 0.078 |  |  |
|  |  | Faecalibacterium | MR-Egger | 5.43 | 0.711 | -0.0009 | 0.783 |
|  |  |  | IVW | 5.51 | 0.788 |  |  |
|  |  | Lachnospiraceae UCG001 | MR-Egger | 19.44 | 0.054 | -0.0039 | 0.583 |
|  |  |  | IVW | 20.00 | 0.067 |  |  |
|  |  | Oscillibacter | MR-Egger | 14.84 | 0.251 | -0.0009 | 0.837 |
|  |  |  | IVW | 14.89 | 0.314 |  |  |
|  |  | Ruminiclostridium9 | MR-Egger | 8.75 | 0.271 | -0.0024 | 0.729 |
|  |  |  | IVW | 8.91 | 0.350 |  |  |
|  |  | Ruminococcus1 | MR-Egger | 17.87 | 0.022 | -0.0005 | 0.928 |
|  |  |  | IVW | 17.89 | 0.036 |  |  |
|  |  | Veillonella | MR-Egger | 7.97 | 0.240 | -0.0060 | 0.436 |
|  |  |  | IVW | 8.90 | 0.260 |  |  |

**Table S5 Sensitivity analysis of IGF-1 on significant gut microbiota in men and women.**

| **Exposure** | **Sex** | **Outcome** | **Heterogeneity test** | | | **Pleiotropy test** | |
| --- | --- | --- | --- | --- | --- | --- | --- |
|  |  |  | **Method** | **Cochran’s Q** | **Cochran’s P** | **Egger_intercept** | **P-Egger intercept** |
| **IGF-1** | Men | **Order** |  |  |  |  |  |
|  |  | Actinomycetales | MR-Egger | 102.45 | 0.875 | -0.0047 | 0.283 |
|  |  |  | IVW | 103.62 | 0.871 |  |  |
|  |  | **Family** |  |  |  |  |  |
|  |  | Acidaminococcaceae | MR-Egger | 111.08 | 0.730 | 0.0011 | 0.756 |
|  |  |  | IVW | 111.18 | 0.749 |  |  |
|  |  | Actinomycetaceae | MR-Egger | 102.38 | 0.749 | -0.0049 | 0.268 |
|  |  |  | IVW | 103.62 | 0.876 |  |  |
|  |  | **Genus** |  |  |  |  |  |
|  |  | Actinomyces | MR-Egger | 106.42 | 0.807 | -0.0058 | 0.196 |
|  |  |  | IVW | 108.11 | 0.793 |  |  |
|  |  | Eisenbergiella | MR-Egger | 116.25 | 0.296 | -0.0015 | 0.766 |
|  |  |  | IVW | 116.33 | 0.554 |  |  |
|  |  | Eubacterium nodatum group | MR-Egger | 136.89 | 0.080 | 0.0081 | 0.274 |
|  |  |  | IVW | 138.32 | 0.077 |  |  |
|  |  | Eubacterium xylanophilum group | MR-Egger | 137.14 | 0.165 | -0.0010 | 0.779 |
|  |  |  | IVW | 137.23 | 0.180 |  |  |
|  |  | Lachnospiraceae ND3007 group | MR-Egger | 123.39 | 0.473 | -0.0033 | 0.268 |
|  |  |  | IVW | 124.63 | 0.467 |  |  |
|  |  | Ruminococcus gauvreauii group | MR-Egger | 143.88 | 0.096 | 0.0018 | 0.606 |
|  |  |  | IVW | 144.19 | 0.104 |  |  |
|  | Women | **Genus** |  |  |  |  |  |
|  |  | Butyricicoccus | MR-Egger | 130.06 | 0.507 | 0.0007 | 0.787 |
|  |  |  | IVW | 130.13 | 0.530 |  |  |
|  |  | Ruminococcaceae UCG014 | MR-Egger | 130.68 | 0.491 | 0.0013 | 0.609 |
|  |  |  | IVW | 130.94 | 0.510 |  |  |


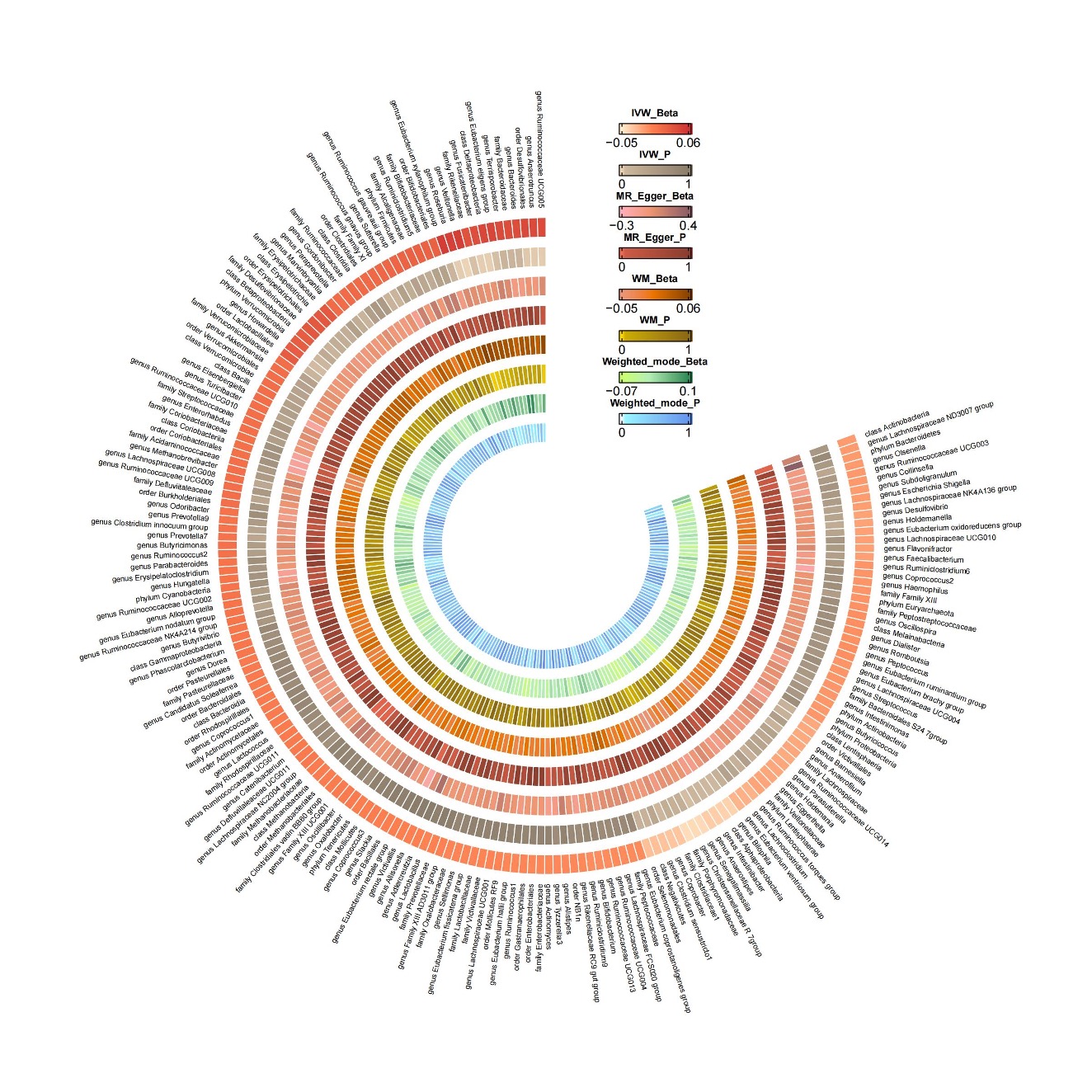


**Figure S1.** Causal effect of gut microbiome on IGF-1 in men. From the outside to the inside are beta and P values indicating IVW, MR Egger, WM, and weighted mode, respectively. IVW, inverse variance weighted; WM, weighted median.


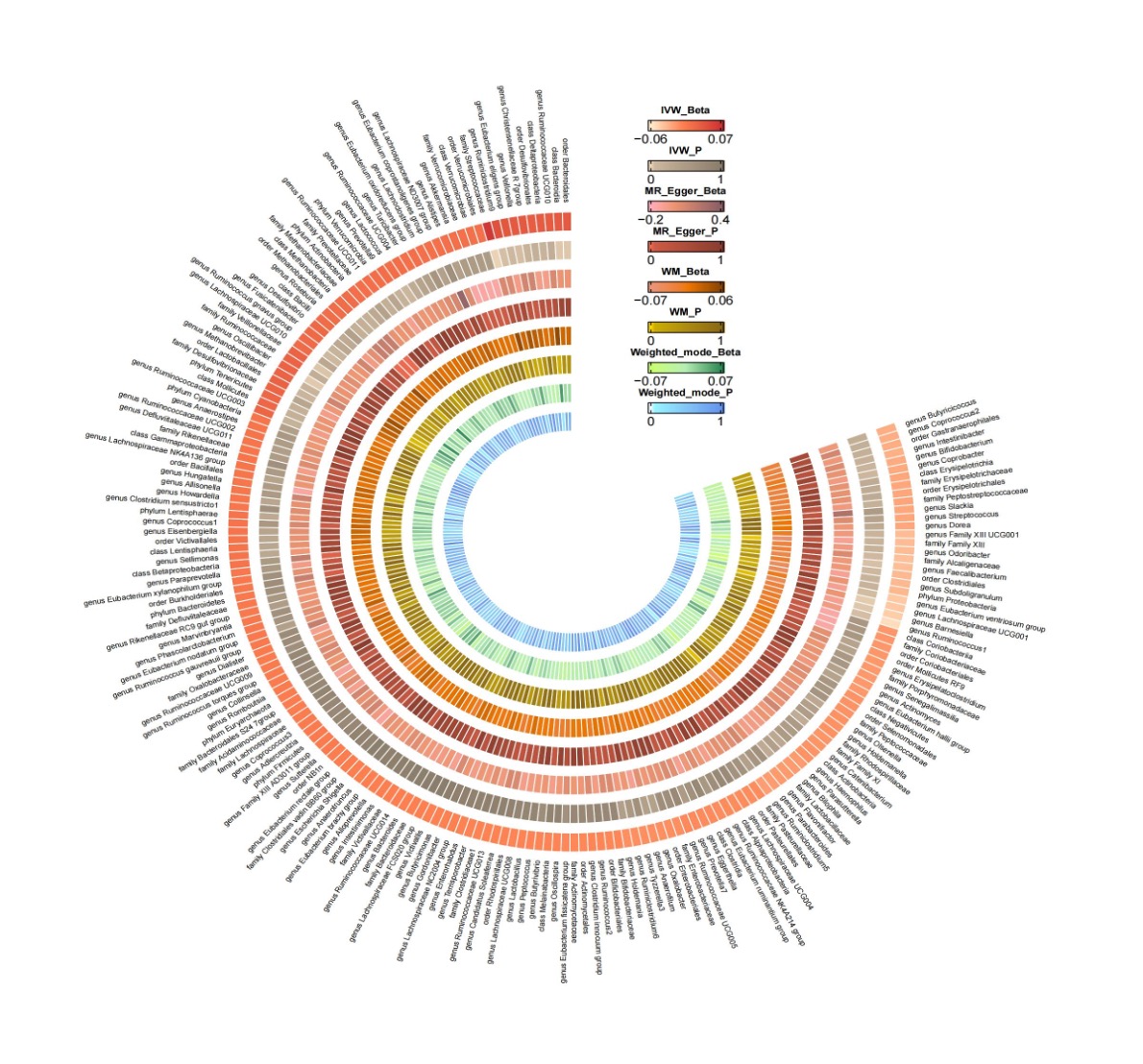


**Figure S2.** Causal effect of gut microbiome on IGF-1 in women. From the outside to the inside are beta and P values indicating IVW, MR Egger, WM, and weighted mode, respectively. IVW, inverse variance weighted; WM, weighted median.


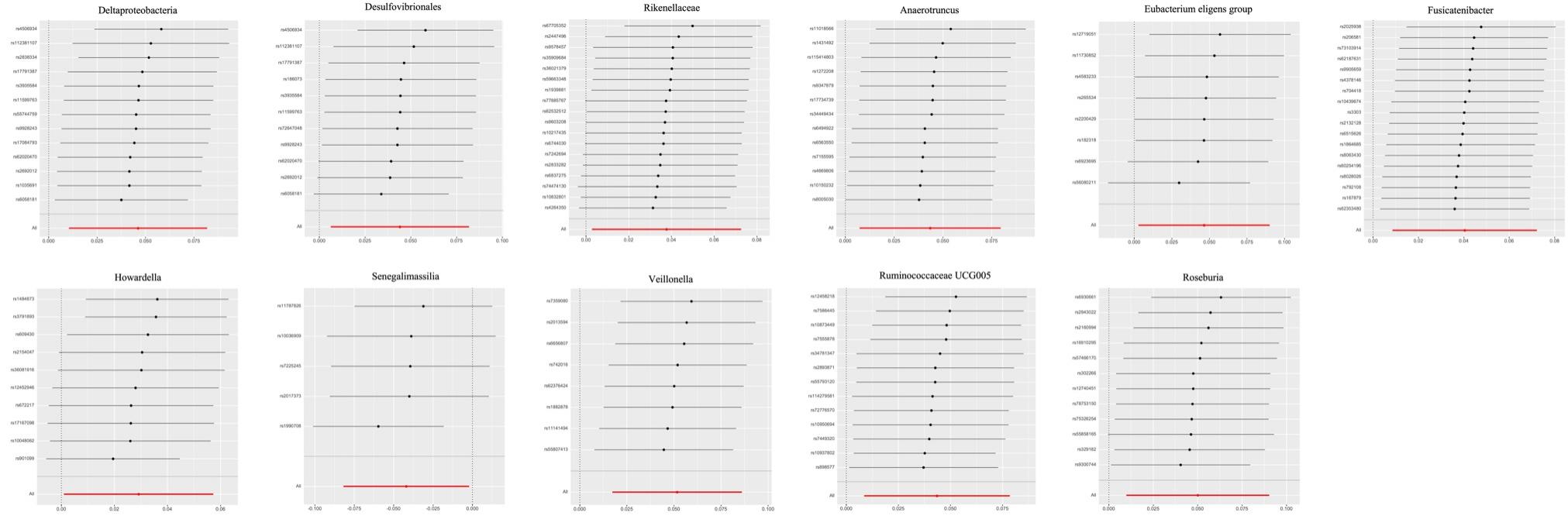


**Figure S3.** The leave-one-out analysis of gut microbiome on IGF-1 in men.


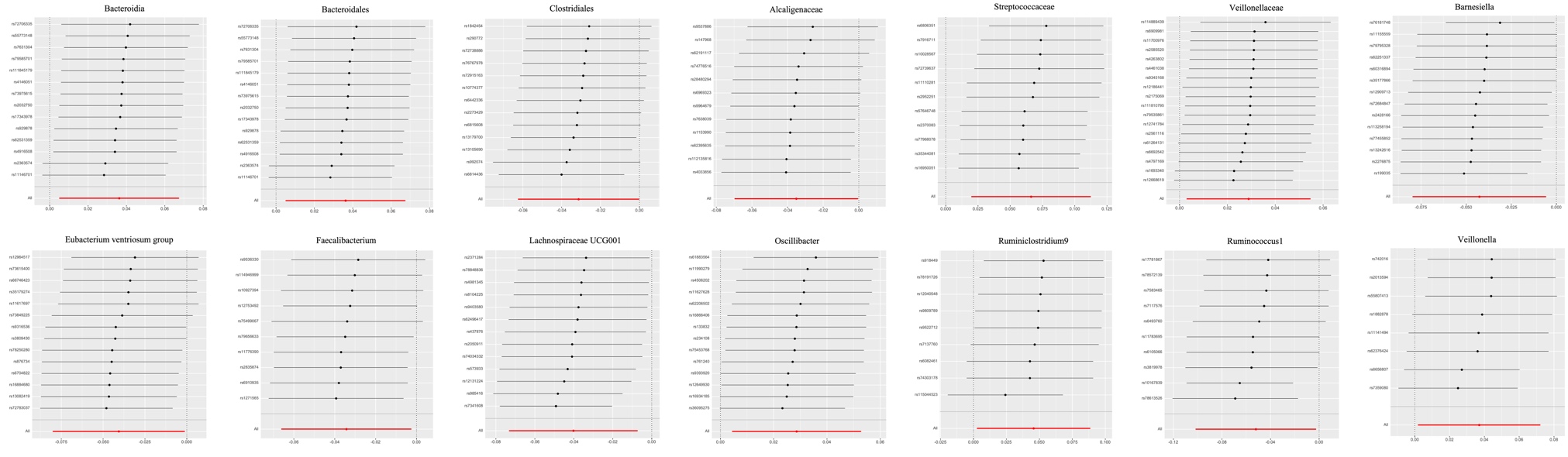


**Figure S4.** The leave-one-out analysis of gut microbiome on IGF-1 in women.


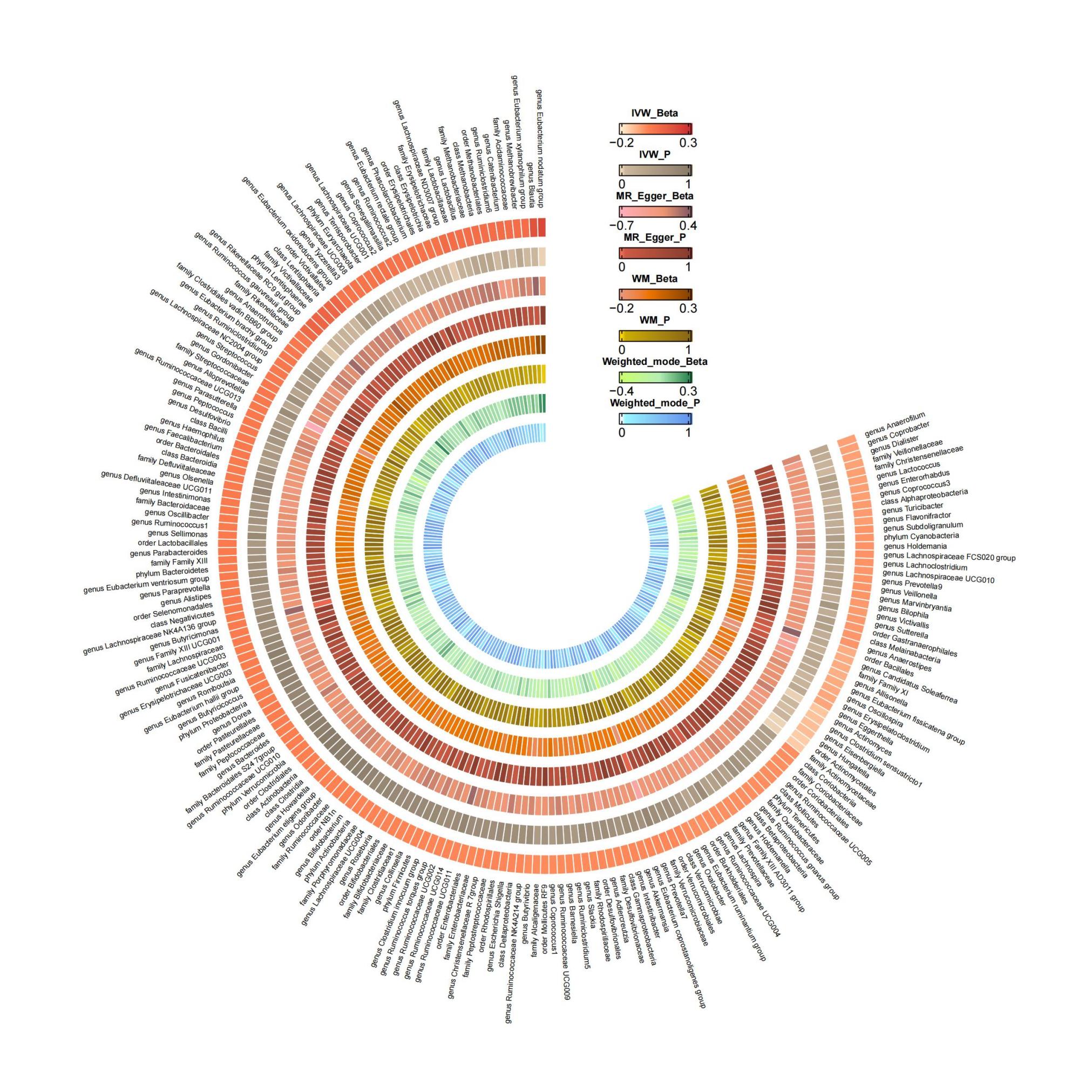


**Figure S5.** Causal effect of IGF-1 on gut microbiome in men. From the outside to the inside are beta and P values indicating IVW, MR Egger, WM, and weighted mode, respectively. IVW, inverse variance weighted; WM, weighted median.


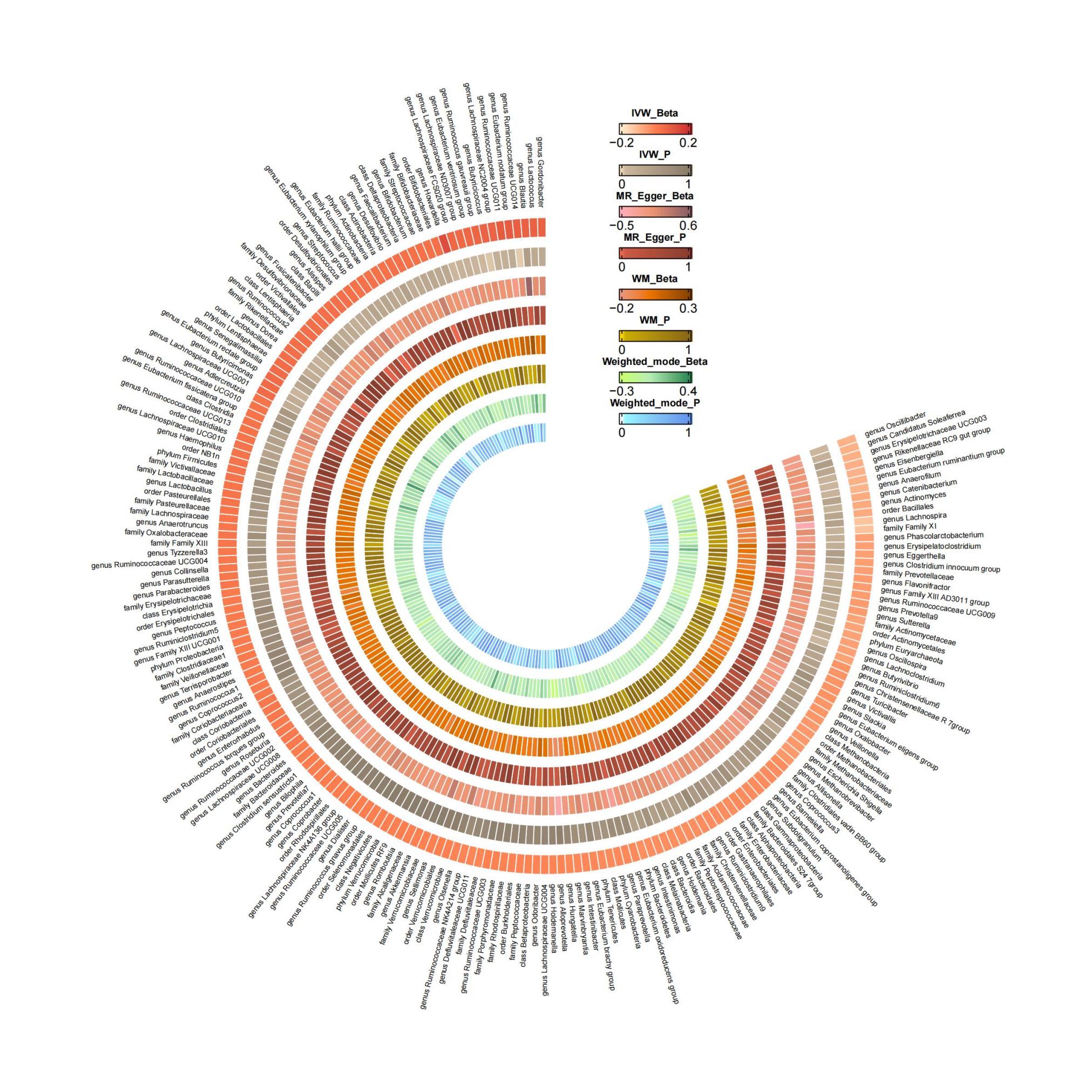


**Figure S6.** Causal effect of IGF-1 on gut microbiome in women. From the outside to the inside are beta and P values indicating IVW, MR Egger, WM, and weighted mode, respectively. IVW, inverse variance weighted; WM, weighted median.
