# Supplementary material for: Causal relationship between gut microbiota and insulin-like growth factor 1: a bidirectional two-sample Mendelian randomization study
Source: Front Cell Infect Microbiol. 2024 Sep 24;14:1406132. doi: 10.3389/fcimb.2024.1406132 (PMC11463061; doi:10.3389/fcimb.2024.1406132)
Supplement: SUPPLEMENTARY DATA SHEET 1 — Code and comments for MR analysis. [file DataSheet1.docx]

**This is the code of the literature "Causal Relationship Between Gut Microbiota and Insulin-like Growth Factor 1: A bidirectional two-sample Mendelian randomization study", all running on R.**

**The software required to run the code and its version was R (version 4.2.0).**

#--------------Causal Effects of Gut Microbiota on IGF-1:---------------

#------------------A Two-sample Mendelian Randomization Study-------------------

#Load the relevant functions

library(gwasglue)

library(gwasvcf)

library(VariantAnnotation)

library(TwoSampleMR)

library(dplyr)

library(data.table)

#1 Read the exposure variable

#1.1 Set the working directory

setwd("/Users/yuyangzhang/Desktop/Mendelian")

#1.2 Read the exposure variable

exposure <- fread("")

#1.3 Correlation Setup: Use the subset function to select SNPs (P < 1e-05)

exposre_p <- subset(exposure,p<1e-05)

#1,4 Independence Setup: Read the data, then apply the clump function

exposure_p <- system.file("exposre_p.csv",package = "TwoSampleMR")

exposure_p_exp_data <- read_exposure_data(filename = exposure_p,sep = ",", snp_col = "SNP",

beta_col = "beta",se_col = "se",eaf_col = "eaf",

effect_allele_col = "effect_allele",other_allele_col = "other_allele",

pval_col ="p",clump = TRUE)

exposure_p_exp_dat_clumped <- clump_data(dat =exposure_p_exp_dat,clump_r2 = 0.001,clump_kb = 10000,

clump_p1 = 1,clump_p2 = 1,pop = "EUR")

#2 Read the outcome variable

#2.1 Set the working directory

setwd("/Users/yuyangzhang/Desktop/Mendelian")

outcome <- fread("",header = TRUE)

#2.2 Merge the exposure and outcome variables using the merge function

data <- merge(exposure_p_exp_dat_clumped,outcome,by.x="SNP",by.y="SNP")

write.csv(data,file = "outcome.csv")

#2.3 Read the outcome variable

outcome_dat <- read_outcome_data("outcome.csv",snps = bmi_exp_data$SNP,sep = ","

,snp_col = "SNP",filename = "outcome.csv",beta_col = "beta"

,se_col = "se",pval_col = "p",effect_allele_col = "effect_allele"

,other_allele_col = "other_allele")

#2.4 Use the harmonize_data function for data harmonization

data_final <- harmonise_data(exposure_dat = bmi_exp_data,outcome_dat = outcome_dat)

write.csv(data_final,file = "data_final.csv")

#3 Mendelian randomization analysis

#3.1 MR analysis

mr(data_final)

#3.2 MR analysis_OR

generate_odds_ratios(mr_res = mr(data_final))

#3.3 MR analysis_scatter plot

mr_scatter_plot(mr_results = mr(data_final),dat = data_final)

#4 Conduct sensitivity analysis

#4.1 Perform heterogeneity testing

mr_heterogeneity(dat = data_final)

mr_funnel_plot(singlesnp_results = mr_singlesnp(data_final))

#4.2 MR_PRESSO

run_mr_presso(dat = data_final,NbDistribution = 3000)

#4.3 Conduct pleiotropy testing

mr_pleiotropy_test(data_final)

#4.4 leave-one-out analysis

mr_leaveoneout(data_final)

mr_leaveoneout_plot(leaveoneout_results = mr_leaveoneout(data_final))
