# Supplementary material for: Causal relationship between gut microbiota and insulin-like growth factor 1: a bidirectional two-sample Mendelian randomization study
Source: Front Cell Infect Microbiol. 2024 Sep 24;14:1406132. doi: 10.3389/fcimb.2024.1406132 (PMC11463061; doi:10.3389/fcimb.2024.1406132)
Supplement: SUPPLEMENTARY DATA SHEET 2 — STROBE-MR-checklist. [file DataSheet2.pdf]

STROBE-MR checklist of recommended items to address in reports of Mendelian randomization studies

Item No.	Section	Checklist item	Page No.	Relevant text from manuscript
1	**TITLE and ABSTRACT**	Indicate Mendelian randomization (MR) as the study's design in the title and/or the abstract if that is a main purpose of the study	1	Causal Relationship Between Gut Microbiota and Insulin-like Growth Factor 1: A bidirectional two-sample Mendelian randomization study<br>Abstract<br>Background: The causal relationship between gut microbiota and insulin-like growth factor 1 (IGF-1) remains unclear. The purpose of this study was to explore the causal relationship between gut microbiota and IGF-1 in men and women.<br>Methods: Single nucleotide polymorphisms (SNPs) related to gut microbiota were derived from pooled statistics from large genome-wide association studies (GWAS) published by the MiBioGen consortium. Pooled data for IGF-1 were obtained from a large published GWAS. We conducted Mendelian Randomization (MR) analysis, primarily using the Inverse Variance Weighted (IVW) method. Additionally, we performed sensitivity analyses to enhance the robustness of our results, focusing on assessing heterogeneity and pleiotropy.<br>Results: In forward MR analysis, 11 bacterial taxa were found to have a causal effect on IGF-1 in men; 14 bacterial taxa were found to have a causal effect on IGF-1 in women (IVW, all $P<0.05$). After FDR correction, all bacterial traits failed to pass the FDR correction. In reverse MR analysis, IGF-1 had a causal effect on 9 bacterial taxa in men, and 2 bacterial taxa in women respectively (IVW, all $P<0.05$). After FDR correction, the causal effect of IGF-1 on order Actinomycetales ($P_{FDR}=0.049$) remains in men. The robustness of the IVW results was further confirmed after heterogeneity and pleiotropy analysis.

				Conclusion: Our study demonstrates a bidirectional causal link between the gut microbiota and IGF-1, in both men and women.
	**INTRODUCTION**			
2	**Background**	Explain the scientific background and rationale for the reported study. What is the exposure? Is a potential causal relationship between exposure and outcome plausible? Justify why MR is a helpful method to address the study question	2	However, most of the current studies are observational and cannot provide further evidence of a causal relationship between the gut microbiota and IGF-1.<br>A causal relationship implies a direct influence of one variable on another, which is critical for understanding the underlying mechanisms. Mendelian randomization (MR) is a robust and effective method that uses genetic variants (single-nucleotide polymorphisms, SNPs) as instrumental variables to explore the causal effects of the gut microbiome on IGF-1. MR leverages the random assortment of genes at conception, which mimics the randomization process in controlled trials, thus helping to infer causality rather than mere association. Many previous studies have described the principles of MR and its reliability. MR has been widely used to explore the causal relationship between exposure and disease and has been used several times in studies of the relationship between gut microbiota and disease.
3	**Objectives**	State specific objectives clearly, including pre-specified causal hypotheses (if any). State that MR is a method that, under specific assumptions, intends to estimate causal effects	2	The goals of this study were to explore the possible causal relationship between gut microbiota and IGF-1 by MR analysis, and to determine if this relationship is bidirectional, spanning both men and women populations. By understanding these causal links, we aim to provide insights into how modulating gut microbiota can influence IGF-1 levels and vice versa, potentially offering new therapeutic targets for related diseases.
	**METHODS**			
4	**Study design and data sources**	Present key elements of the study design early in the article. Consider including a table listing sources of data for all phases of the study. For each data source contributing to the analysis, describe the following:		

a)	Setting: Describe the study design and the underlying population, if possible. Describe the setting, locations, and relevant dates, including periods of recruitment, exposure, follow-up, and data collection, when available.		
b)	Participants: Give the eligibility criteria, and the sources and methods of selection of participants. Report the sample size, and whether any power or sample size calculations were carried out prior to the main analysis	2-3	Data sources of gut microbiome<br>The causal relationship between gut microbiota and IGF-1 was assessed by a bidirectional two-sample MR, and the study design and flowchart were shown in Figure 1. The MiBioGen consortium published a large-scale gene-wide association study (GWAS) of the composition of the gut microbiota. This dataset contained a total of 18,340 samples of 16S rRNA gene sequencing data from 24 population-based cohorts. A total of 211 gut microbiomes were identified from genus to phylum. Subsequently, 15 bacteria were excluded due to unknown traits, and finally 119 genera, 32 families, 20 orders, 16 classes, and 9 phyla were included in the MR analysis. The original article described more detailed information about the gut microbiome.<br>Data sources of IGF-1<br>Sex-specific datasets on IGF-1 were derived from the UK Biobank (http://www.nealelab.is/uk-biobank), utilizing GWAS summary statistics involving 361,194 participants of European ancestry. Principal component analysis (PCA) was performed on the genetic data to adjust for population stratification, and the top 20 principal components were included as covariates in the analysis. These principal components capture the major axes of genetic variation within the dataset, thus controlling for potential confounding due to population structure. Genetic associations were also adjusted for age and age squared (age²), expressed as per standard deviation changes in IGF-1.
c)	Describe measurement, quality control and selection of genetic variants	3	Genetic instrument selection<br>In this study, we followed strict criteria to ensure the robustness and validity of this MR study. For the gut microbiota as exposure, in order to obtain sufficient SNPs to be used as instrumental variables (IVs), a P-value of <1e-5 was set as the

					significance threshold to select genetic instruments associated with bacterial traits. This threshold ensured that the selected SNPs are strongly associated with the exposure, reducing the risk of weak instrument bias. We set the chain imbalance threshold r^2 to <0.001 and the distance to 10,000 kb to avoid this phenomenon of linkage disequilibrium (LD). This process minimized the inclusion of correlated SNPs that might confound the MR analysis. IVs of the gut microbiota were shown in Table S1. In addition, we used the MR Pleiotropy RESidual Sum and Outlier (MR-PRESSO) method to look for significant SNPs with pleiotropy and excluded outliers if present. MR-PRESSO identified and removed significant outliers that might introduce pleiotropy, ensuring that the causal estimates were not biased by pleiotropic effects. The results of the F-statistic represent the strength of the IVs (F-statistics = $Beta^2/Se^2$, beta is the correlation coefficient between SNPs and traits), and SNPs with F-statistic values > 10 indicate that there is no substantial weak instrumental bias, otherwise the IVs were removed. Strong instruments were crucial for reliable causal inference in MR studies. For our study, all F-statistics were greater than 10.
		d)	For each exposure, outcome, and other relevant variables, describe methods of assessment and diagnostic criteria for diseases		
		e)	Provide details of ethics committee approval and participant informed consent, if relevant		
5	**Assumptions**		Explicitly state the three core IV assumptions for the main analysis (relevance, independence and exclusion restriction) as well assumptions for any additional or sensitivity analysis	3	MR analysis must meet the following three assumptions to be performed correctly: (1) the assumption of relevance: the IVs used for the analysis should be closely related to the exposure; (2) the assumption of independence: the IVs were not related to the exposure or confounders of the outcome; and (3) the assumption of exclusivity: the IVs were not related to the outcome.

6	**Statistical methods: main analysis**		Describe statistical methods and statistics used	4	We used the inverse variance weighting (IVW) approach as the primary method to explore the causal relationship between exposure and outcome, an analysis that has the advantage of providing robust causality estimates in the absence of directional pleiotropy (consistent with the independence assumption). In addition, three other additional analytical methods including MR-Egger, weighted median analysis (WM), and weighted mode method were conducted as secondary references to improve the reliability of causality. The MR-Egger method was based on the assumption that all IVs are invalid and the intercept term is present by default.The WM analysis method was based on the assumption that more than 50% of SNPs have valid SNPs. For estimation methods based on weighted models, smaller sample sizes are required, but lower type-I error rates and smaller biases are guaranteed. P < 0.05 indicates statistical significance. We also adjusted for the results in multiple comparisons (Benjaminiand Hochberg) by false discovery rate (FDR).
		a)	Describe how quantitative variables were handled in the analyses (i.e., scale, units, model)		
		b)	Describe how genetic variants were handled in the analyses and, if applicable, how their weights were selected		
		c)	Describe the MR estimator (e.g. two-stage least squares, Wald ratio) and related statistics. Detail the included covariates and, in case of two-sample MR, whether the same covariate set was used for adjustment in the two samples		
		d)	Explain how missing data were addressed		
		e)	If applicable, indicate how multiple testing was addressed		
7	**Assessment of assumptions**		Describe any methods or prior knowledge used to assess the assumptions or justify their validity	4	The MR-Egger method was based on the assumption that all IVs are invalid and the intercept term is present by default.The WM analysis method was based on the assumption that more than 50% of SNPs have valid SNPs. For estimation methods based on weighted models, smaller sample sizes are required, but lower type-I error rates and smaller biases are guaranteed. P < 0.05 indicates statistical significance.
8	**Sensitivity analyses and additional analyses**		Describe any sensitivity analyses or additional analyses performed (e.g. comparison of effect estimates from different approaches, independent replication, bias analytic techniques, validation of instruments, simulations)	4	The purpose of the sensitivity analysis was to test for heterogeneity and horizontal pleiotropy of IVs in MR analysis. We performed Cochran's Q test for heterogeneity of IVs with MR-Egger and IVW methods. Heterogeneity could indicate that the instruments were not consistently estimating the same causal effect, which could bias the results. Cochran's Q test compared the observed variance among the effect estimates of the genetic instruments to what would be expected if

				all instruments were estimating the same effect. A P-value greater than 0.05 from Cochran's Q test indicated that there was no significant heterogeneity among the IVs, suggesting that the instruments are homogeneous and the causal estimates are reliable. In addition, horizontal pleiotropy was assessed using two methods: MR Egger intercept and MR-PRESSO global test. The MR Egger regression method provides an intercept term that can be used to test for directional pleiotropy. A significant non-zero intercept indicates the presence of pleiotropy, which can bias the MR estimates. In our analysis, a p-value greater than 0.05 for the intercept term suggests that there is no evidence of horizontal pleiotropy, indicating that the genetic instruments are not affecting the outcome through pathways other than the exposure. MR-PRESSO global test method is used to detect and correct for horizontal pleiotropy. This method identifies significant outliers that may contribute to pleiotropy. The global test within MR-PRESSO assesses the overall pleiotropy by comparing the observed data to the expected distribution under no pleiotropy. A p-value greater than 0.05 indicates the absence of significant pleiotropy. Furthermore, MR-PRESSO analysis reveals specific SNP outliers that contribute to pleiotropy, which can be removed to refine the causal estimates and reduce bias. To further validate the stability of our results, we performed a "Leave-One-Out" analysis, which evaluates the influence of each individual SNP on the overall causal estimate. Of course, we used PhenoScanner (BMI and smoking status) to exclude potentially pleiotropic SNPs that were significantly associated with confounding factors (http://www.phenoscanner.medschl.cam.ac.uk/).
9	**Software and pre-registration**			
		a) Name statistical software and package(s), including version and settings used	4	All MR analyses in this study were performed in the R software (version 4.2.0, The R Foundation,

				Vienna, Austria) with the "TwoSampleMR", "MR-PRESSO", "frostplot" and "ggplot2" packages.
		b) State whether the study protocol and details were pre-registered (as well as when and where)		
	**RESULTS**			
10	**Descriptive data**			
		a) Report the numbers of individuals at each stage of included studies and reasons for exclusion. Consider use of a flow diagram	4	Figure 1
		b) Report summary statistics for phenotypic exposure(s), outcome(s), and other relevant variables (e.g. means, SDs, proportions)		
		c) If the data sources include meta-analyses of previous studies, provide the assessments of heterogeneity across these studies		
		d) For two-sample MR:<br>i. Provide justification of the similarity of the genetic variant-exposure associations between the exposure and outcome samples<br>ii. Provide information on the number of individuals who overlap between the exposure and outcome studies		
11	**Main results**			
		a) Report the associations between genetic variant and exposure, and between genetic variant and outcome, preferably on an interpretable scale		Additional file 1,2
	b)	Report MR estimates of the relationship between exposure and outcome, and the measures of uncertainty from the MR analysis, on an interpretable scale, such as odds ratio or relative risk per SD difference		Causal effects of the gut microbiome on IGF-1 in MR analysis<br>The research design and flow chart of this study was shown in Figure 1. A total of 196 bacterial traits from five biological levels (phylum, order, family, and genus) were finally included in this study. In the forward MR analysis, the causal effects of 196 bacterial taxa on IGF-1 in men and women were shown in Figs. S1-S2, respectively. As shown in Table 1, genetically predicted class.Deltaproteobacteria (beta=0.046, 95% CI=0.010 to 0.082, P=0.011), order.Desulfovibrionales (beta=0.044, 95% CI=0.006 to 0.082, P=0.022), family.Rikenellaceae (beta=0.038, 95% CI=0.003 to 0.073, P=0.034), genus.Anaerotruncus

(beta=0.044, 95% CI=0.007 to 0.080, P=0.018), genus.Eubacterium eligens group (beta=0.046, 95% CI=0.003 to 0.090, P=0.037), genus. Fusicatenibacter (beta=0.040, 95% CI=0.009 to 0.072, P=0.013), genus.Howardella (beta=0.029, 95% CI=0.001 to 0.057, P=0.043), genus. Senegalimassilia (beta=-0.042, 95% CI=-0.082 to -0.002, P=0.039), genus. Veillonella (beta=0.052, 95% CI=0.017 to 0.086, P=0.003), genus.Ruminococcaceae UCG005 (beta=0.044, 95% CI=0.009 to 0.079, P=0.015), and genus.Roseburia (beta=0.050, 95% CI=0.010 to 0.090, P=0.015) had a causal effect on IGF-1 in men (Figure 2A), whereas genetically predicted class.Bacteroidia (beta=0.036, 95% CI=0.005 to 0.067, P=0.023), order.Bacteroidales (beta=0.036, 95% CI=0.005 to 0.067, P=0.023), order.Clostridiales (beta=--0.031, 95% CI=-0.062 to -0.000, P=0.049), family.Alcaligenaceae (beta=-0.035, 95% CI=-0.070 to -0.000, P=0.048), family. Streptococcaceae (beta=-0.066, 95% CI=0.020 to 0.112, P=0.005), family.Veillonellaceae (beta=-0.029, 95% CI=0.003 to 0.055, P=0.029), genus.Barnesiella (beta=-0.043, 95% CI=-0.080 to -0.006, P=0.024), genus.Eubacterium ventriosum group (beta=-0.041, 95% CI=-0.080 to -0.001, P=0.044), genus.Faecalibacterium (beta=-0.034, 95% CI=-0.066 to -0.002, P=0.035), genus.Lachnospiraceae UCG001 (beta=-0.040, 95% CI=-0.073 to -0.007, P=0.017), genus.Oscillibacter (beta=0.029, 95% CI=0.004 to 0.053, P=0.021), genus. Ruminiclostridium9 (beta=0.046, 95% CI=0.003 to 0.089, P=0.037), genus. Ruminococcus1 (beta=-0.052, 95% CI=-0.102 to -0.002, P=0.040), and genus.Veillonella (beta=0.037, 95% CI=0.002 to 0.072, P=0.039) had a causal effect on IGF-1 in women (Figure 2B). Overall, our forward MR analysis identified 11 bacterial taxa with a causal effect on IGF-1 in men and 14 in women. Compared to observational

				studies, our study provides stronger causal inferences.<br>In the forward MR analysis, all bacterial traits failed to pass the FDR correction (P>0.05). In the sensitivity analysis, Cochran's Q test showed no sign of heterogeneity for all bacterial traits (Table S4). No pleiotropy was found in the results by MR-Egger and MR-PRESSO analytical methods (Table S4). In addition, the results of leave-one-out analysis provide further evidence of the robustness of the results (Figure S3-S4).
	c)	If relevant, consider translating estimates of relative risk into absolute risk for a meaningful time period		
	d)	Consider plots to visualize results (e.g. forest plot, scatterplot of associations between genetic variants and outcome versus between genetic variants and exposure)		Figure 2-3
12	Assessment of assumptions			
	a)	Report the assessment of the validity of the assumptions	5	In the forward MR analysis, all bacterial traits failed to pass the FDR correction (P>0.05). In the sensitivity analysis, Cochran's Q test showed no sign of heterogeneity for all bacterial traits (Table S4). No pleiotropy was found in the results by MR-Egger and MR-PRESSO analytical methods (Table S4). In addition, the results of leave-one-out analysis provide further evidence of the robustness of the results (Figure S3-S4).
	b)	Report any additional statistics (e.g., assessments of heterogeneity across genetic variants, such as I2, Q statistic or E-value)		
13	Sensitivity analyses and additional analyses		5<br>Additional file 2	In the forward MR analysis, all bacterial traits failed to pass the FDR correction (P>0.05). In the sensitivity analysis, Cochran's Q test showed no sign of heterogeneity for all bacterial traits (Table S4). No pleiotropy was found in the results by MR-Egger and MR-PRESSO analytical methods (Table S4). In addition, the results of leave-one-out analysis provide further evidence of the robustness of the results (Figure S3-S4).
	a)	Report any sensitivity analyses to assess the robustness of the main results to violations of the assumptions		
	b)	Report results from other sensitivity analyses or additional analyses		
	c)	Report any assessment of direction of causal relationship (e.g., bidirectional MR)		

	d)	When relevant, report and compare with estimates from non-MR analyses		After FDR correction, the causal effect of IGF-1 on order.Actinomycetales (P_{FDR}=0.049) remained in men. Figure 4 further demonstrate**d** the stability of this result**.** In the sensitivity analysis, the results of Cochran's Q test showed no signs of heterogeneity for all bacterial traits (Table S5). No pleiotropy was found in the results by MR-Egger and MR-PRESSO analytical methods (Table S5). In addition, the results of leave-one-out analysis provided further evidence of the robustness of the results (Table S6-S7).
	e)	Consider additional plots to visualize results (e.g., leave-one-out analyses)		
	DISCUSSION			
14	Key results	Summarize key results with reference to study objectives	6	In this MR study, this study is the first to investigate the causal relationship between gut microbiota IGF-1 through large-scale GWAS summary data. Our findings provide new insights into the complex interplay between gut microbiota and growth factors, which have significant implications for understanding metabolic and endocrine disorders.<br>Our study corroborates these findings and further elucidates the directionality of these relationships. We demonstrated that certain bacterial taxa causally influence IGF-1 levels and vice versa. Specifically, our forward MR analysis identified 11 bacterial taxa with a causal effect on IGF-1 in men and 14 in women. Compared to observational studies, our study provides stronger causal inferences.<br>On this basis, the results of our reverse MR analysis demonstrated that IGF-1 has a causal effect on 9 bacterial taxa in men, and 2 bacterial taxa in women respectively. After FDR correction, the causal effect of IGF-1 on order. Actinomycetales (P_{FDR}=0.049) remained in men.
15	Limitations	Discuss limitations of the study, taking into account the validity of the IV assumptions, other sources of potential bias, and imprecision. Discuss both direction and magnitude of any potential bias and any efforts to address them	7-8	This study, while offering valuable insights, is not without its limitations. Firstly, it's important to note that the genetic data pooled from GWAS primarily included European participants, which may restrict the applicability of our findings to other populations, thereby constraining the

generalizability of our results. Secondly, our analytical scope was constrained by the capabilities of our classifiers and sequencing methods, which permitted us to examine the gut microbiota only at the genus level and higher taxonomic classifications. Thirdly, the inherent limitations of the available data prevented us from assessing individual-level associations. Additionally, although our MR approach provides robust causal inference, several potential confounders and biases, including population stratification, measurement error, and reverse causation, still need to be considered. Population stratification can introduce bias if there are systematic differences in allele frequencies between subpopulations with different ancestries. IGF-1 data source was adjusted for the top 20 principal components in the analysis, which capture the major axes of genetic variation and help control for population structure. Despite these adjustments, residual confounding due to population stratification may still exist and should be acknowledged. In our study, we used well-established GWAS data from large consortia (MiBioGen and UK Biobank), which have rigorous quality control measures to minimize measurement error. However, it is important to recognize that residual measurement error cannot be entirely ruled out, and such errors may attenuate the estimated causal effects. Our bidirectional MR approach helps mitigate this concern by examining the causal effects in both directions. Nonetheless, it is essential to interpret the findings in the context of potential reverse causation and consider additional evidence from experimental or longitudinal studies to strengthen the causal inference. Other important confounding factors, such as overall health status, nutritional status, and detailed dietary habits, could not be explored in our analysis due to data limitations. Lastly, the MR itself has limitations, as it does not take into account epigenetic modifications, which

				may affect gene expression and IGF-1 production. The MR assumes a direct causal pathway, oversimplifying the complex biological processes involved, such as the roles of bacterial metabolites, inflammation, and nutrient absorption. Additionally, although we used stringent criteria to select genetic instruments, residual pleiotropy could still bias our results. Further studies with larger sample sizes are needed to confirm these findings.
16	Interpretation			
	a)	Meaning: Give a cautious overall interpretation of results in the context of their limitations and in comparison with other studies	8	Our study demonstrates a bidirectional causal link between the gut microbiota and IGF-1, spanning both men and women populations. Even after correction, most of the results became non-significant, but could still suggest an association between gut microbiota and IGF-1. This discovery implies that the associated microbiota are potential therapeutic targets for promoting homeostasis of these hormones, which in turn may modulate gut microbiota homeostasis.
	b)	Mechanism: Discuss underlying biological mechanisms that could drive a potential causal relationship between the investigated exposure and the outcome, and whether the gene-environment equivalence assumption is reasonable. Use causal language carefully, clarifying that IV estimates may provide causal effects only under certain assumptions	7	Many different bacterial species can regulate IGF-1 levels, so the common production of a microbial metabolite by these species, short-chain fatty acids (SCFA), has the potential to provide an explanation for the mechanism by which the gut microbiota regulates host IGF-1. SCFA, including acetate, propionate, and butyrate, represent a wide range of microbial metabolites generated through the fermentation of non-digestible dietary fibers. Among these, butyrate stands out as the primary source of energy for enterocytes. SCFA can exert their effects both within the local environment of the intestinal tract and systemically by entering the bloodstream. SCFA concentrations in the feces of conventionally reared mice were higher than those in germ-free mice. In a study by Yan J et al., conventional mice treated with broad-spectrum antibiotics and vancomycin experienced a reduction in SCFA concentrations, while the cecum of colonized

			germ-free mice exhibited increased SCFA levels. Additionally, as cecal SCFA concentrations roughly correlated with trends in serum IGF-1, they demonstrated that, akin to colonization in mice, supplementation with SCFA led to enhanced production of IGF-1 in adipose tissue and a noticeable trend toward increased IGF-1 production in the liver. This suggests that the gut microbiota may influence IGF-1 production either directly or indirectly through the generation of SCFA. A comprehensive review supports the link between microbially produced SCFA and IGF-1, reporting that feeding non-digestible fiber, oligosaccharides (fermented into SCFA), and probiotics promotes bone health. However, it is not possible to definitively conclude that SCFA **we**re sufficient to directly induce IGF-1, and it **wa**s likely that additional microbiota–host interactions contribute to the increased IGF-1 production by host tissues. SCFA may indirectly influence the production of IGF-1 through the following mechanisms: SCFA can improve insulin sensitivity, thereby enhancing the anabolic effects of insulin and subsequently influencing the production of IGF-1; SCFA can strengthen gut barrier integrity, supporting the nutrient absorption necessary for IGF-1 synthesis; SCFA can regulate lipid metabolism, maintaining lipid balance, which is important for metabolic health and can indirectly affect IGF-1.
c)	Clinical relevance: Discuss whether the results have clinical or public policy relevance, and to what extent they inform effect sizes of possible interventions	7	The bidirectional causal relationship between gut microbiota and IGF-1 has important implications for understanding the underlying mechanisms of metabolic and endocrine disorders. For instance, the identified causal effects of gut microbiota on IGF-1 levels suggest potential therapeutic targets for modulating IGF-1 levels through microbiota interventions. Conversely, understanding how IGF-1 influences gut microbiota composition could lead to novel strategies for managing conditions such as obesity, diabetes, and growth disorders. Our findings also underscore the importance of

				considering both directions of causality in future research to fully capture the complexity of these interactions.
17	**Generalizability**	Discuss the generalizability of the study results (a) to other populations, (b) across other exposure periods/timings, and (c) across other levels of exposure	7	Our study corroborates these findings and further elucidates the directionality of these relationships. We demonstrated that certain bacterial taxa causally influence IGF-1 levels and vice versa. Specifically, our forward MR analysis identified 11 bacterial taxa with a causal effect on IGF-1 in men and 14 in women. Compared to observational studies, our study provides stronger causal inferences.
	**OTHER INFORMATION**			
18	**Funding**	Describe sources of funding and the role of funders in the present study and, if applicable, sources of funding for the databases and original study or studies on which the present study is based	8	Funding<br>None
19	**Data and data sharing**	Provide the data used to perform all analyses or report where and how the data can be accessed, and reference these sources in the article. Provide the statistical code needed to reproduce the results in the article, or report whether the code is publicly accessible and if so, where		
20	**Conflicts of Interest**	All authors should declare all potential conflicts of interest	8	Conflicts of Interest:<br>The authors declare that they have no conflict of interest.

This checklist is copyrighted by the Equator Network under the Creative Commons Attribution 3.0 Unported (CC BY 3.0) license.

1. Skrivankova VW, Richmond RC, Woolf BAR, Yarmolinsky J, Davies NM, Swanson SA, et al. Strengthening the Reporting of Observational Studies in Epidemiology using Mendelian Randomization (STROBE-MR) Statement. JAMA. 2021;under review.

2. Skrivankova VW, Richmond RC, Woolf BAR, Davies NM, Swanson SA, VanderWeele TJ, et al. Strengthening the Reporting of Observational Studies in Epidemiology using Mendelian Randomisation (STROBE-MR): Explanation and Elaboration. BMJ. 2021;375:n2233.
